# Neurocognitive Mechanisms of Preventive Cognitive Therapy as Relapse Prevention in Remitted Major Depressive Disorder: A Randomized Controlled Trial

**DOI:** 10.1016/j.bpsgos.2026.100781

**Published:** 2026-06-25

**Authors:** Marie-José van Tol, Rozemarijn van Kleef, Ronja Eike, Evelien van Valen, Jan-Bernard Marsman, Remco Renken, André Aleman, Claudi Bockting

**Affiliations:** a Center for Clinical Neuroscience and Cognition, University Medical Center Groningen, Groningen, the Netherlands; b Research School for Behavioural and Cognitive Neurosciences, University of Groningen, Groningen, the Netherlands; c Department of Medical Psychology, University Medical Center Groningen, Groningen, the Netherlands; d Department of Geriatrics, University Medical Center Utrecht, Utrecht University, Utrecht, the Netherlands; e Faculty of Psychology and Neurosciences, Maastricht University, Maastricht, the Netherlands; f Department of Psychiatry, University Medical Center Amsterdam, Amsterdam, the Netherlands; g Center for Urban Mental Health, University of Amsterdam, Amsterdam, the Netherlands

**Keywords:** Emotion regulation, fMRI, Major depressive disorder, Neurocognitive mechanisms, Preventive cognitive therapy, Relapse prevention

## Abstract

**Background:**

Major depressive disorder (MDD) is a highly recurrent psychiatric disorder. Understanding mechanisms of effective treatments that lower recurrence risk provides an opportunity to identify neurocognitive targets for recurrence prevention. Here we studied neurocognitive mechanisms of preventive cognitive therapy (PCT).

**Methods:**

Fifty medication-free individuals who were remitted from MDD and at high risk for recurrence were randomized to 8 sessions of protocolized PCT or a waiting list control (WLC) group as part of the NEWPRIDE (Neurocognitive Working Mechanisms of the Prevention of Relapse in Depression) study (NTR5368). Individuals were assessed at baseline and a 3-month follow-up using functional magnetic resonance imaging, cognitive testing, and clinical questionnaires. Primary outcomes were changes in 1) prefrontal cortex activation during effortful emotion regulation and 2) biased automatic processing, covering positive and negative affective dimensions. Secondary outcomes included changes in symptomatology, cognitive and affective reactivity, and emotion regulation styles. Linear mixed models and repeated-measures analyses of variance were conducted to test for PCT-induced changes.

**Results:**

Following PCT (vs. WLC), decreased (dorso)medial prefrontal activation during effortful regulation of positive affect and decreased pregenual anterior cingulate activation during effortful regulation of positive and negative affect were observed, alongside increased reactivity of positive self-related thinking and lower negative affect responsivity.

**Conclusions:**

Results from this primary analysis show that PCT, which is a nonpharmacological preventive intervention, has neuromodulatory effects on the prefrontal brain circuitry that underpin the regulation of affect and boost positive thinking about oneself, which is associated with lower negative affect. Training the affect and beliefs to more positive content may strengthen the neural circuitry that is implicated in guarding against the activation of negative affect and beliefs in the face of daily negative events, thereby lowering the risk for recurrence.

## Plain Language Summary

Depression often recurs after recovery. In this study, we tested how preventive cognitive therapy (PCT), a psychological treatment, can help prevent relapse and how it affects the brain. Fifty people who had recovered from depression received either PCT or no treatment. After 3 months, people who had received PCT showed changes in brain areas involved in regulating emotions, as well as more positive thinking about themselves and less negative emotional responses. These changes suggest that PCT may help strengthen regulatory brain processes that protect against future depressive episodes.

Recurrence is a common disruptive and demoralizing feature of major depressive disorder (MDD). Because the risk for recurrence is associated with the number of previous episodes (1), and each depressive episode is highly disruptive to normal occupational, social, and personal functioning (2, 3, 4), preventing MDD recurrence is an urgent clinical goal.

A number of preventive treatment strategies are currently available (5,6). One such treatment is preventive cognitive therapy (PCT), which was specifically designed to prevent relapse during the remitted phase of MDD. PCT is aimed at challenging dysfunctional attitudes and schemas by using positive affect and imagery, enhancing positive autobiographical memories, and designing a personal prevention plan (7,8). PCT has shown long-lasting relapse-preventing effects (up to 20 years) (9, 10, 11, 12), suggesting that it causes enduring mechanistic changes that lower the likelihood of relapse. The mechanisms through which PCT obtains its preventive effects are not fully clear yet (13, 14, 15, 16). However, elucidating those mechanisms will provide important insights into what is needed to lower recurrence risk and optimize treatments toward attaining higher preventive power.

Studies on cognitive therapy (CT) delivered during the acute depressed phase indicate that normalization of attentional biases and use of regulatory strategies when facing negative information are related to treatment success (17). On a neural level, CT has been shown to normalize responsivity of brain regions that are involved in emotional processing and cognitive control (18, 19, 20, 21, 22, 23). This suggests that CT may obtain its remission-promoting effects (24) by increasing engagement of prefrontal brain areas that are involved in the effortful regulation of negative affective states (25, 26, 27). Whether PCT delivered during the remitted phase obtains its preventive effects by similar or additional changes is an open question.

To this end, we set up a randomized controlled trial (RCT) to investigate changes in neurocognitive and cognitive-affective functioning in unmedicated individuals who were remitted from multiple depressive episodes following PCT compared to individuals on a waiting list (waiting list control [WLC] group). Because PCT primarily focuses on using techniques that address positive beliefs and affect, and effects of (P)CT on positive emotion processing have received little attention to date, we studied both valences. Based on neurocognitive frameworks of depression (1,28,29) and proposed relapse-facilitating factors (15,30, 31, 32, 33, 34), we primarily studied immediate changes in brain responsivity during instructed regulation of negative and positive emotions as measured with functional magnetic resonance imaging (fMRI) and biases in processing of negative and positive information. We hypothesized that compared to WLC, PCT would result in increased lateral prefrontal brain activation during instructed emotion regulation and lowered bias toward negative information and away from positive information. Secondarily, we explored effects on dysfunctional automatic thinking, cognitive reactivity and emotion regulation styles, and negative and positive affect and affect reactivity.

## Methods and Materials

### Design and Ethics

The NEWPRIDE (Neurocognitive Working Mechanisms of the Prevention of Relapse in Depression) study is an open-label RCT investigating mechanisms of protocolized PCT for preventing depressive relapse carried out at the University Medical Center Groningen in the Netherlands. The study compared 2 conditions, a PCT condition and a WLC condition. The study protocol, including power calculations and all study aims, has been described in detail elsewhere (35) and was registered in the Netherlands Trial Register (NTR5368/NL-OMON26793). Ethical approval was obtained from the Medical Ethical Committee of the University Medical Center Groningen. All participants were drawn from a community sample and provided written consent. Participants received €75 and reimbursement of travel costs for their participation. PCT was offered free of charge.

### Participants

In total, 75 individuals with remitted recurrent MDD (rrMDD) were included in the NEWPRIDE study between 2016 and 2021. This report focuses on the first 50 participants with rrMDD who were randomized to either PCT (*n* = 25) or WLC (*n* = 25) and describes the immediate changes following therapy. An additional 25 participants with rrMDD all received PCT to allow prediction of longitudinal treatment effects based on pretreatment characteristics, and owing to an unforeseen change of MRI scanner, they were scanned on a different scanner. These participants are not included in analyses testing the primary research question addressed in the current report, following the strategy outlined in the protocol paper (35).

### Inclusion Criteria

Individuals were included who 1) were between 18 and 60 years old; 2) showed normal intelligence; 3) fulfilled criteria for at least 2 major depressive episodes (MDEs) during the past 5 years; 4) were in remission for at least 2 months but no longer than 2 years; 5) scored ≤13 on the Inventory of Depression Symptomatology (IDS) (36), indicative of no current depressive symptomatology; 6) used no psychotropic medication for at least 4 weeks; 7) received no protocolized cognitive (behavioral) therapy for their last episode; 8) did not fulfill criteria for any other current Axis I psychiatric disorder according to DSM-IV; 9) reported no past alcohol or drug dependency according to DSM-IV; and 10) had no general MRI contraindications.

### Procedure, Randomization, and Masking

Included individuals participated in a baseline (T0) measurement, after which they were randomized to 8 sessions of PCT or WLC using a block-randomization procedure. Participants in the WLC were informed that they could start PCT following the 3-month follow-up (T1) measurement. Finally, a clinical examination was performed at an 18-month follow-up (T2). See Supplement 1 for the Preferred Reporting Items for Systematic Reviews and Meta-Analyses (PRISMA) flow chart.

Because our aim in this study is to understand what changes following PCT in individuals with rrMDD, participants who dropped out before or during the intervention phase or who scored ≥21 on the IDS at the time of the baseline measurement were not included in the analyses and were replaced if possible (35).

### Intervention

PCT was delivered by experienced and licensed psychologists, who were fully trained in cognitive behavioral therapy (CBT) and who had received an additional 2-day training in providing PCT in the context of the NEWPRIDE study (EvV, CB). Substantial measures were taken to establish adequate treatment integrity. See Supplement 2 for details.

### Outcomes

Table 1 provides an overview of primary and secondary outcome measures assessed at T0 and T1. Primary outcome instruments are described below. See Supplements 3 to 7 for complete descriptions of these instruments.

**Table 1:** List of Primary and Secondary Outcome Measures

Instrument	Measure	Index
Primary Outcomes		
ERT-fMRI	Brain activation during downregulation of negative emotions	Activation during negative reappraisal vs. negative processing
	Brain activation during upregulation of positive emotions	Activation during positive reappraisal vs. positive processing
	Affective reactivity & self-rated emotion regulation success	Total VAS scores (affective and performance ratings) following negative and positive processing and reappraisal blocks
ARDPEI	Early attentional bias for negative information	Negative attention bias - engagement
		Negative attention bias - disengagement
	Early attentional bias for positive information	Positive attention bias - engagement
		Positive attention bias - disengagement
IAT	Implicit depressive self-assocations	DnoEP score
EmoReas	Reactivity of automatic self-related thinking	Total scores on negative specific thoughts
		Total scores on negative general thoughts
		Total scores on positive specific thoughts
		Total scores on positive general thoughts
Secondary Outcomes		
IDS30-SR	Depressive symptom severity	Total score on IDS-SR30
DOPS	Hedonic pleasure	Total score on anticipatory pleasure scale
PANAS	Positive and negative affect	Total score on positive affect and negative affect scale
LEIDS-RR	Cognitive reactivity	Total score
LARSS	Rumination on sadness	Total score
RPA	Rumination on positive emotions	Total score
DAS-A	Dysfunctional attitudes	Total score
ERQ	Habitual emotion regulation strategy use in daily life	Total scores on scales CR and AS
BVAQ	Alexithymia	Total scores on scales of cognitive alexithymia and affective alexithymia

See the Supplement for a complete description of the instruments. EmoReas is an adaptation from (66), see Supplement 7 for details.

ARDPEI, Attentional Response to Distal vs. Proximal Emotional Information (64); AS, Affective Suppression; BVAQ, Bermond Vorst Alexithymia Questionnaire (74); CR, cognitive reappraisal; DAS-A, Dysfunctional Attitude Scale – Version A (72); DOPS, Domains of Pleasure Scale (67); EmoReas, Emotional Reasoning Task; ERQ, Emotion Regulation Questionnaire (73); ERT-fMRI, Emotion Regulation Task – administered during fMRI (63); IAT, Implicit Association Test (65); IDS 30-SR, Inventory of Depressive Symptomatology, 30 item self-report version (36); LARSS, Leuven Adaptation on the Rumination on Sadness Scale (70); LEIDS-RR, Leiden Index of Depressive Sensitivity – 2nd Revision (69); PANAS, Positive and Negative Affect Schedule (68); RPA, Responses to Positive Affect Scale (71).

### Primary Outcome Measures

#### Brain Activation During Emotion Regulation

All participants performed an Emotion Regulation Task (ERT) (37) (Supplement 4) during 3T fMRI, including a regulate and attend instruction. The task was used to calculate regional brain activation differences between the following conditions: negative downregulation > negative attend and positive upregulation > positive attend (see Supplement 8). During the ERT-fMRI session, the pupil dilation responses were also recorded using an SR Research MR-compatible Eyelink system at a sampling rate of 250 Hz.

#### Emotional Bias Processing

We used an adapted version of the Attentional Response to Distal Versus Proximal Emotional Information (ARDPEI) task (Supplement 5) (38), which is an adaptation of the classical dot-probe task to measure differentiation between engagement and disengagement biases in response to briefly (500 ms and 1000 ms) presented visual stimuli (see 37).

#### Implicit Association Test

A computerized Implicit Association Test (IAT) (25) (see Supplement 6) was administered to assess the strength of implicit negative self-associations participants may have regarding themselves. The IAT measures whether a person has more automatic self-associations with positive or negative attributes based on response latencies, which is indexed by calculating a DnoEP score [based on correct trials only, consistent with (25)]. Higher IAT scores indicate stronger negative self-associations.

#### Emotional Reasoning Task

Reactivity of automatic negative and positive thoughts was assessed with the Emotional Reasoning Task (19) (see Supplement 7), which is a computerized task in which participants rated the likelihood of certain specific and general self-related thoughts that arise in response to 12 positive and 12 negative scenarios, half of them followed by a description (cue) of how the situation would make them feel (i.e., “you feel happy” or “you feel sad”).

### Statistical Analysis

Questionnaire and behavioral data were analyzed with JASP (version 0.19.0.0, JASP team, University of Amsterdam). fMRI data were analyzed using 3dLME tools (39) implemented in AFNI (40). See Supplement 8 for full details on preprocessing and first-level modeling.

### Primary Outcome Measures

#### fMRI Analysis: LME Modeling

To test for time × treatment condition effects on the contrast maps reflecting regulation of positive and negative images versus attendance, 3 linear mixed effects models (LMEs) were set up.

The first LME included condition (2: PCT, WLC) as a between-subjects fixed factor and time (2: baseline, follow-up) and valence (2: negative, positive) as within-subject factors. The contrast images negative downregulate > negative attend and positive upregulation > positive attend were entered as dependent variables. We modeled the time × condition interaction in this model. Next, we set up 2 LMEs including condition (2: PCT, WLC) as a between-subjects fixed factor and time (2: baseline, follow-up) as a within-subject factor separately for negative downregulate > negative attend and positive upregulation > positive attend.

#### fMRI Analysis: Statistical Testing

fMRI effects were considered significant at *p* < .05, familywise error rate-corrected at the cluster level (see Supplement 9).

#### ERT Affect and Performance Ratings

We set up repeated-measures analyses of variance (RM ANOVAs) with condition (2: PCT, WLC) as a between-subjects factor and time (2: baseline, follow-up) and instruction (2: regulate and attend) as within-subject factors to test for the effect on affect ratings. Performance ratings were only investigated for the regulation condition. We set up separate models for positive and negative valences.

#### ARDPEI Task

We set up 2 RM ANOVAs with condition (2: PCT, WLC) as a between-subjects factor and time (2: baseline, follow-up), direction (2: engagement, disengagement), and duration (2: 2500 ms, 1000 ms) as within-subject factors to test for effects on positive and negative attentional biases separately. We report on the effects of condition × time × direction across durations.

#### Implicit Association Test

We set up an RM ANOVA with condition (2: PCT, WLC) as a between-subjects factor and time (2: baseline, follow-up) as a within-subject factor to test for the effect on automatic self-associations with positive or negative attributes.

#### Emotional Reasoning

We set up 4 RM ANOVAs with condition (2: PCT, WLC) as a between-subjects factor and time (2: baseline, follow-up) and cue (no/yes) as within-subject factors to test for the effect of treatment on self-related thinking following positive and negative scenarios for the specific and general thoughts separately. We report on the effects of condition × time × cue and condition × time per valence category (positive and negative).

For all RM ANOVAs, interactions were considered significant at *p* < .05. Post hoc explorations were performed to follow-up significant effects or effects of the RM ANOVAs that showed at least a moderate effect size (η_p_^2^ > 0.06). We used Wilcoxon signed-rank tests that were considered significant at *p* < .05 (2-sided) and survived false discovery rate (FDR) correction for the number of post hoc comparisons, following the Benjamini-Hochberg procedure (Data S1) with *q* < .05, indicated as *q*_*wsrt*_.

### Secondary Outcome Measures

We set up an RM ANOVA per outcome measure (see Table 1) to test for the interaction of time (2: baseline, follow-up) × condition (2: PCT, WLC). Interactions were considered significant at *p* < .05, FDR-corrected for the number of secondary outcome measures explored (*k* = 12) using the Benjamini-Hochberg procedure (Data S1). FDR thresholds of *q* < .05 and *q* < .15 are both reported (which we deemed warranted given the exploratory nature of the outcome measures), indicated as *q*_rm−anova_; post hoc explorations were further FDR-corrected at *q* < .05, following the Benjamin-Hochberg-procedure, for the number of paired *t* tests that were performed (indicated as *q*_*wsrt*_).

## Results

### Sample Descriptives

Twenty-five individuals with rrMDD were randomized to WLC, and 25 were randomized to PCT, 6 of whom did not complete the treatment; 3 of these noncompleters could be replaced, and 3 of them could not be replaced because the inclusion period had ended. The final analysis includes data from 47 individuals with rrMDD who participated in both T0 and T1 measurements. High-quality MRI data were available for 41 participants (20 PCT, 21 WLC) for T0 and 37 participants (16 PCT, 21 WLC) for T1. Individuals in the 2 conditions were very similar on demographic and clinical characteristics (Table 2). At T1, 4 patients in the WLC condition had experienced recurrence but none in the PCT condition had (likelihood ratio = 5.377, *p* = .020). See Supplement 10 for a full overview of descriptives per outcome measure. Because of the low proportion of pupil dilation datasets recorded during fMRI that were of sufficiently high quality and the high number of trials that had to be removed, we could not calculate pupil dilation responses for positive and negative regulation conditions separately. Therefore, results of the pupil dilation responses are not included in this report.

**Table 2:** Demographic and Clinical Characteristics

	Group	*N*	Mean (SD)	Range
Age, Years	WLC	25	35.440 (12.490)	18.000–55.000
	PCT	22	36.364 (9.892)	21.000–55.000
Sex, Female/Male	WLC	25	21/4	–
	PCT	22	15/7	–
Education, Years	WLC	24	16.250 (3.554)	6.000–24.000
	PCT	21	17.667 (2.477)	13.000–23.000
Handedness, Left/Right	WLC	25	22/3	–
	PCT	22	19/3	–
IQ Score	WLC	23	97.696 (9.227)	82.000–116.000
	PCT	17	97.118 (7.793)	84.000–111.000
Depression History				

Age of onset, last episode, years	WLC	25	34.520 (12.437)	16.000–54.000
	PCT	22	35.091 (9.875)	20.000–53.000
Duration last episode, months	WLC	25	6.740 (4.255)	0.500–18.000
	PCT	22	6.750 (4.956)	0.500–22.000
Age of onset, forelast episode, years	WLC	25	32.200 (12.884)	14.000–52.000
	PCT	22	32.909 (10.932)	12.000–53.000
Duration forelast episode, months	WLC	25	8.280 (8.542)	1.000–36.000
	PCT	21	10.048 (14.891)	1.000–72.000
Age of onset, first episode, years	WLC	25	23.440 (10.697)	10.000–50.000
	PCT	22	25.045 (11.445)	7.000–51.000
Average duration last 2 episodes, months	WLC	25	7.510 (5.444)	1.500–20.500
	PCT	21	8.036 (8.197)	2.000–41.000
Number of MDEs, lifetime	WLC	25	6.200 (9.657)	2.000–50.000
	PCT	22	6.727 (9.696)	2.000–48.000
Recency last MDE, months	WLC	24	8.167 (6.411)	2.000–21.000
	PCT	21	10.333 (5.782)	1.000–24.000
Past presence of anxiety disorder, no/yes	WLC	25	19/6	–
	PCT	22	20/2	–
Age of onset anxiety disorder, years	WLC	25	43.750 (5.500)	36.000–49.000
	PCT	22	22.500 (17.678)	10.000–35.000
Childhood Trauma Questionnaire score	WLC	25	43.012 (15.368)	26.000–91.000
	PCT	22	38.287 (8.700)	24.000–51.000
Medical and Therapeutic History				
Physical illness, no/yes	WLC	25	22/3	–
	PCT	22	16/6	–
Contraceptive use, no/yes; for women only	WLC	20	15/5	–
	PCT	14	9/5	–
Antidepressant use in the past, no/yes	WLC	25	12/13	–
	PCT	22	9/13	–
SSRI use in the past, no/yes	WLC	25	15/10	–
	PCT	22	14/8	–
Benzodiazepine use in the past, no/yes	WLC	25	23/2	–
	PCT	22	17/5	–
Present medication use, non-AD; no/yes	WLC	25	18/7	–
	PCT	22	16/6	–
CBT use in the past, no/yes	WLC	8	8/17	–
	PCT	5	5/17	–
Other nonpharmacological treatment in the past, no/yes	WLC	21	21/4	–
	PCT	22	22/0	–
1st degree family history, no/yes	WLC	25	15/10	–
	PCT	22	15/7	–
2nd degree family history, no/yes	WLC	25	15/10	–
	PCT	22	16/6	–

AD, antidepressant; CBT, cognitive behavioral therapy; MDE, major depressive episode; PCT, preventive cognitive therapy; SSRI, selective serotonin reuptake inhibitor; WLC, waiting list control.

### Effects of PCT on Primary Outcome Parameters

#### fMRI Correlates of Effortful Emotion Regulation

**Figure 1:**
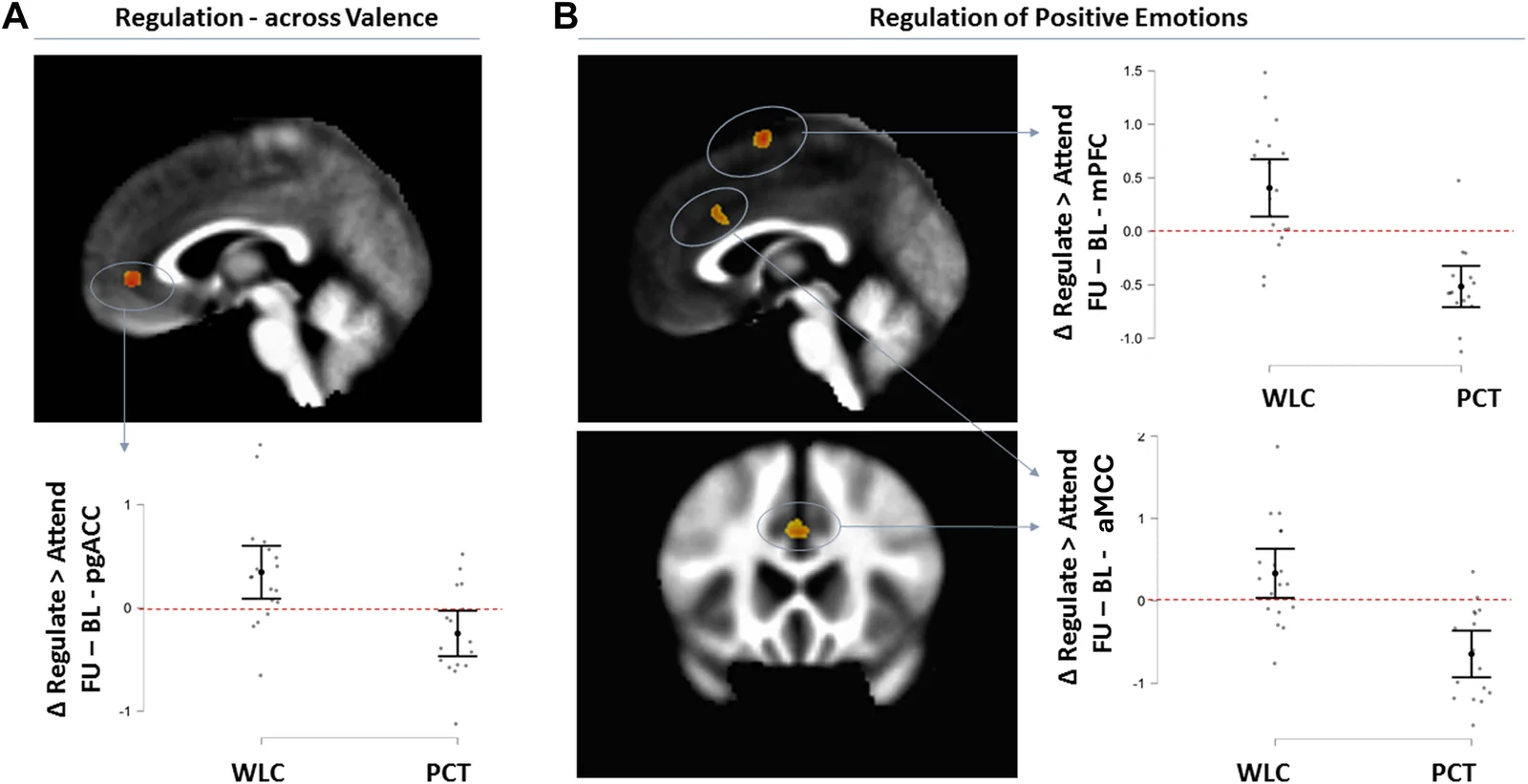
Effects of treatment on brain activation during effortful regulation of emotions. **(A)** Interaction effect of condition (preventive cognitive therapy [PCT] vs. waiting list control [WLC]) on the main effect of the contrast regulate vs. attend across positive and negative task conditions in the pregenual anterior cingulate cortex (pgACC). Plots indicate a small increase in the activation difference between regulate and attend over time (follow-up 1 [FU] minus baseline [BL]) in the WL condition, whereas the PCT condition shows a decrease in the activation difference for regulate vs. attend. **(B)** Interaction effect of condition (PCT vs. WLC) on main effect of the contrast regulate positive vs. attend positive in the medial prefrontal cortex (mPFC) (top display and graph) and the anterior mid-cingulate cortex (aMCC) (both displays and bottom graph). Over time, the WL condition showed either stable or increased activation differences between regulate positive and attend positive for the mPFC and aMCC, respectively, whereas the PCT group showed a decrease in the activation difference between regulate positive and attend positive over time. See Supplement 11 for the main task effects.

Across negative and positive emotion regulation, a significant time × condition effect was observed in the pregenual anterior cingulate cortex (pgACC) (Montreal Neurological Institute [MNI] coordinates: [x = 3; y = 44; z = −4], *k* = 22, *F* = 16.78). While WLC showed an increased activation difference in regulating versus attending over time in the pgACC, PCT showed a decrease (Figure 1A).

During downregulation of negative emotions (vs. attending), no time × condition interaction was observed. During upregulation of positive emotions (vs. attending), a significant time × condition interaction was observed in the anterior mid-cingulate cortex (aMCC; MNI coordinates: [x = 0; y = 23; z = 32], *k* = 20, *F* = 19.14) and in the medial frontal gyrus (MNI coordinates: [x = 0; y = −1; z = 68], *k* = 41, *F* = 29.21). Plots indicated an increase in activation difference between regulation and attend in WLC and a decrease in PCT (Figure 1B). No other effects were observed.

#### Subjective Ratings

**Figure 2:**
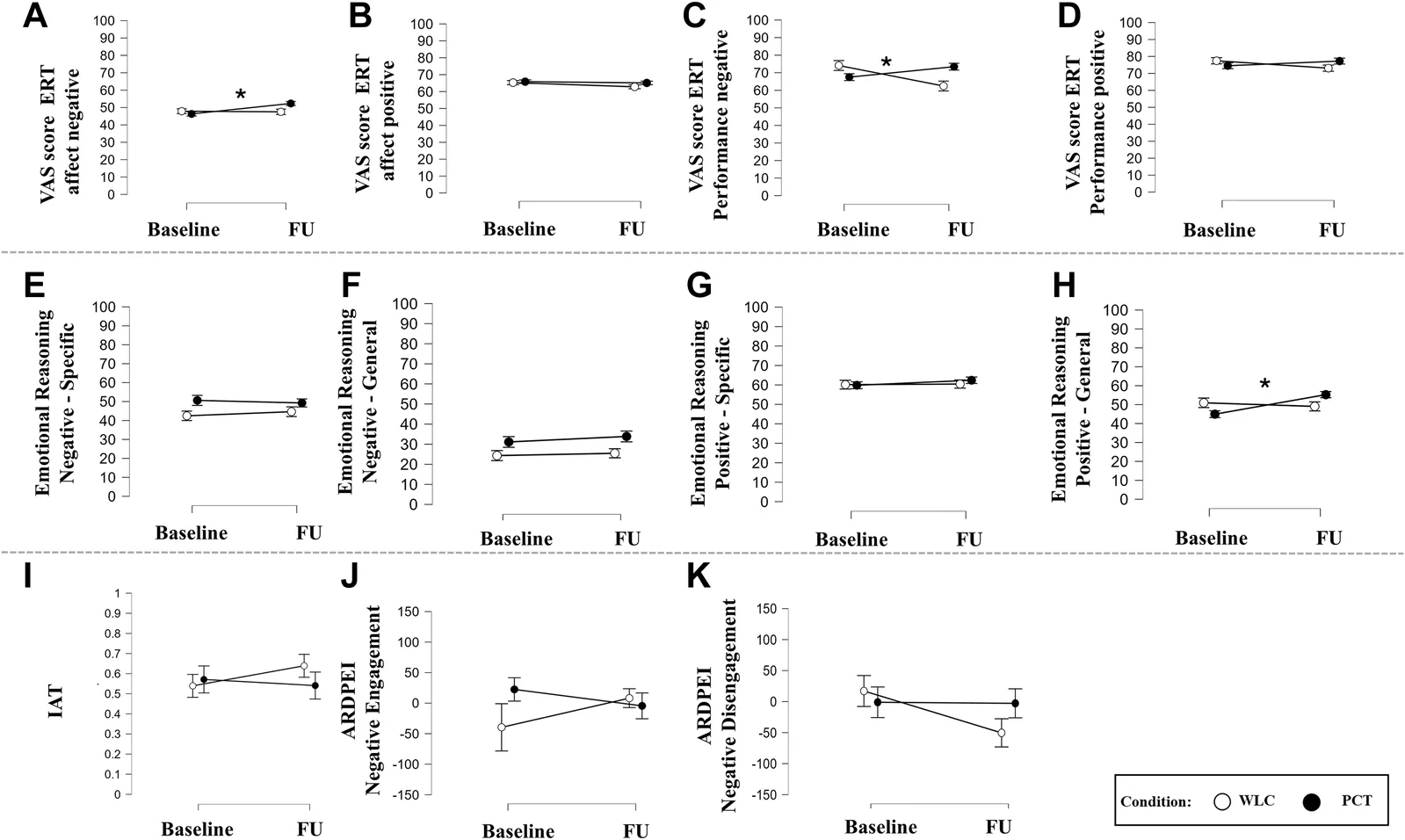
Treatment effects on the primary outcome parameters. Plots (mean ± standard error) depicting the time (baseline vs. follow-up [FU]) × condition (preventive cognitive therapy [PCT] vs. waiting list control [WLC] condition) interaction effects on **(A)** affective ratings as measured using a visual analog scale (VAS) ranging from 0 (very sad) to 100 (very happy) following negative blocks, across attend and regulate instructions; **(B)** affective ratings as measured using a VAS ranging from 0 (very sad) to 100 (very happy) following positive blocks, across attend and regulate instructions; **(C)** performance ratings measured using a VAS ranging from 0 (not at all able to follow the instruction) to 100 (very well able to follow the instructions) following negative regulate blocks; **(D)** performance ratings measured using a VAS ranging from 0 (not at all able to follow the instruction) to 100 (very well able to follow the instructions) following positive regulate blocks; **(E)** emotional reasoning VAS score ranging from 0 (not likely at all) to 100 (very likely) following negative scenarios for specific cognitions; **(F)** emotional reasoning VAS score ranging from 0 (not likely at all) to 100 (very likely) following negative scenarios for general cognitions; **(G)** emotional reasoning VAS score ranging from 0 (not likely at all) to 100 (very likely) following positive scenarios for specific cognitions; **(H)** emotional reasoning VAS score ranging from 0 (not likely at all) to 100 (very likely) following positive scenarios for general cognitions; **(I)** Implicit Association Test (IAT)—DnoEP score; **(J)** Attentional Response to Distal vs. Proximal Emotional Information (ARDPEI) index representing the negative engagement bias; **(K)** ARDPEI index representing the disengagement bias. ∗ indicates a significant interaction of time × treatment condition at *p* < .05.

We observed a time × condition interaction (*F*_1,38_ = 5.114, *p* = .030, η_p_^2^ = 0.119) (Figure 2A) for the affect ratings following negative blocks, reflecting an increase in visual analog scale ratings in the PCT group (indicative of less negative ratings; attending: *z* = −2.286, *p* = .024, *q*_wsrt_ = .048; regulating: *z* = −3.353, *p* = .002, *q*_wsrt_ = .008), while WLC remained stable (attending: *z* = 0.355, *p* = .737, *q*_wsrt_ = .982; regulating: *z* = −0.149, *p* = .896, *q*_wsrt_ = .896). No time × condition × instruction interaction was observed (*F*_1,38_ = 0.048, *p* = .827, η_p_^2^ = 0.001). For the positive blocks, no time × condition (*F*_1,38_ = 0.442, *p* = .510, η_p_^2^ = 0.011) (Figure 2B) or time × condition × instruction interaction was observed (*F*_1,38_ = 1.981, *p* = .167, η_p_^2^ = 0.050).

We observed a time × condition interaction (*F*_1,38_ = 12.734 *p* ≤ .001, η_p_^2^ = 0.251) on emotion regulation performance following negative regulation blocks, reflecting increased self-reported performance in the PCT group (*z* = −2.012, *p* = .045, *q*_*wsrt*_ = .045) (Figure 2C) and decreased performance in the WLC group (*z* = 2.520, *p* = .012, *q*_*wsrt*_ = .024). No time × condition effect was observed following positive regulation blocks, although there was a medium effect size (*F*_1,38_ = 3.895, *p* = .056, η_p_^2^ = 0.093) (Figure 2D).

#### Attentional Biases

A time × condition × direction (engagement vs. disengagement) interaction was observed for negative versus neutral trials (ARDPEI: *F*_1,42_ = 7.097, *p* = .011, η_p_^2^ = 0.145). However, post hoc tests revealed no difference over time in the PCT or WLC group for either engagement or disengagement bias (all *p*s > .05; Figure 2J–K). No other interactions were observed. For the positive versus neutral trials, no effects were observed (*F*s < 2.015, *p*s > .163, η_p_^2^ < 0.046).

No time × condition interaction was observed for the IAT (*F*_1,41_ = 1.027, *p* = .317, η_p_^2^ = 0.024) (Figure 2I).

For the Emotional Reasoning Task (Figure 2E–H), no effect of time × condition or time × condition × cue was observed on specific cognitions following negative and positive scenarios (*F* < 0.593, *p* > .45, η_p_^2^ < 0.014) or on general cognitions following negative scenarios (*F*_1,43_ < 0.114, *p* > .738, η_p_^2^ < 0.003). However, for general cognitions following positive scenarios, a time × condition interaction was observed (*F*_1,44_ = 4.615, *p* = .037, η_p_^2^ = 0.095) (see Figure 2H), but there was no time × condition × cue interaction (*F*_1,44_ = 2.606, *p* = .114, η_p_^2^ = 0.056). Post hoc explorations suggest an increase in general positive cognitions independent of mood induction cue in the PCT group (cue: *z* = −2.659, *p* = .006, *q*_*wsrt*_ = .024; no cue: *z* = −2.694, *p* = .007, *q*_*wsrt*_ = .014), while the WLC group remained stable (cue: *z* = 1.292, *p* = .201, *q*_*wsrt*_ = .268; no cue: *z* = −0.121, *p* = .916, *q*_*wsrt*_ = .916).

### Effects of PCT on Secondary Outcomes

#### Depressive Symptomatology

**Figure 3:**
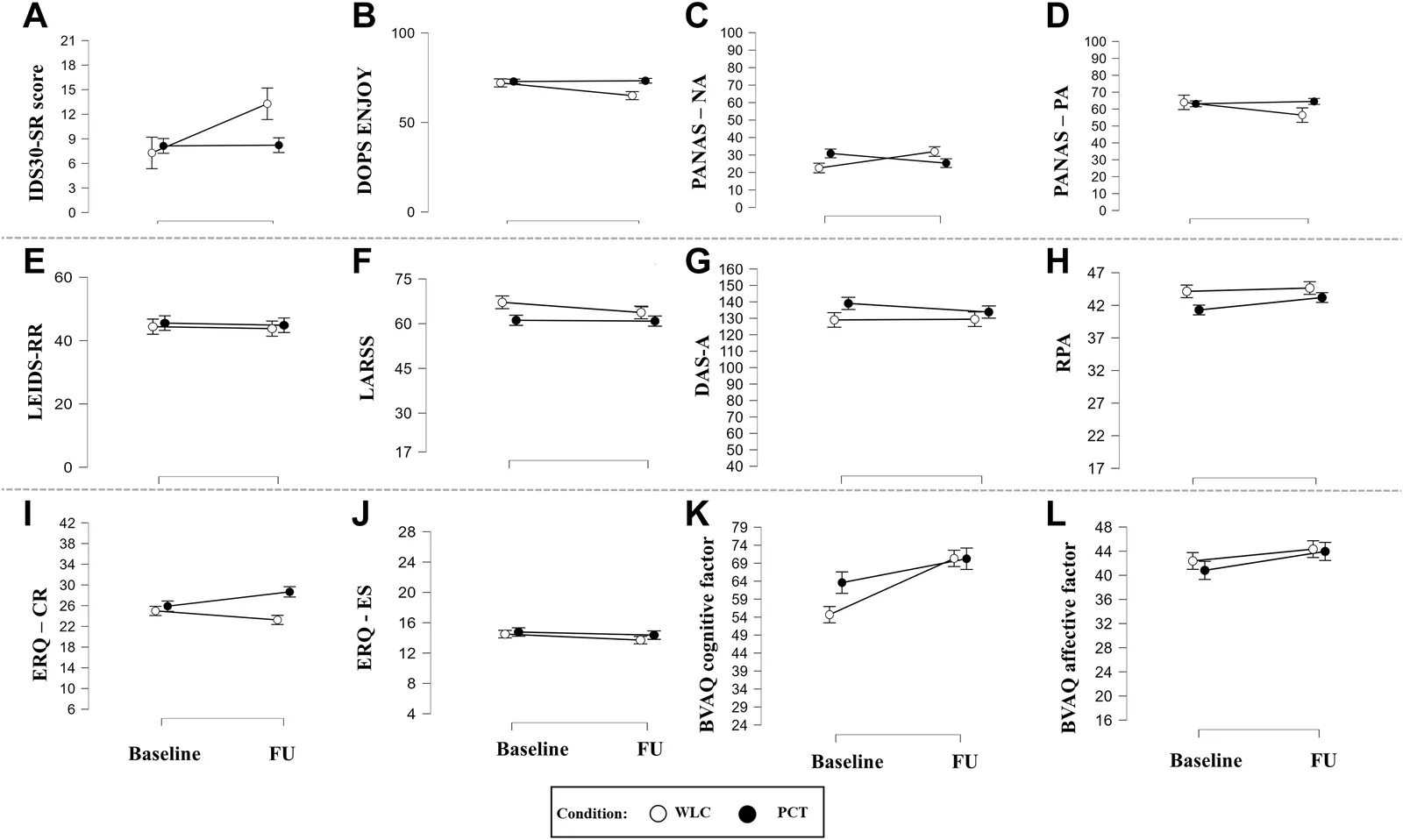
Secondary outcome measures. Plots (mean ± standard error) depicting the time (baseline vs. follow-up [FU]) × condition (preventive cognitive therapy [PCT] vs. waiting list control [WLC]) interaction effects on **(A)** depressive symptomatology as measured by the Inventory of Depressive Symptomatology 30 item self-report (IDS-SR30) version; **(B)** consummatory anhedonia as measured by the Domains of Pleasure Scale (DOPS) enjoy subscale; **(C)** negative affect as measured with the Positive and Negative Affect Schedule (PANAS) negative affect (NA) subscale; **(D)** PANAS positive affect (PA) subscale; **(E)** total score on the Leiden Index of Depressive Sensitivity (LEIDS-RR) – 2nd Revision; **(F)** total score on the Leuven Adaptation of the Rumination on Sadness Scale (LARSS); **(G)** total score on the Dysfunctional Attitude Scale (DAS-A); **(H)** total score on the Responses to Positive Affect (RPA) questionnaire; **(I)** cognitive reappraisal subscale of the Emotion Regulation Questionnaire (ERQ-CR); **(J)** emotional suppression subscale of the Emotion Regulation Questionnaire (ERQ-ES); **(K)** Bermond Vorst Alexithymia Questionnaire (BVAQ) cognitive subscale score; **(L)** BVAQ affective subscale score.

No significant time × condition interactions were observed on IDS 30 item self-report version scores (*F*_1,45_ = 3.538, *p* = .066, η_p_^2^ = 0.073) (Figure 3A) or Domains of Pleasure Scale enjoy scores (*F*_1,44_ = 3.725, *p* = .06, η_p_^2^ = 0.078) (Figure 3B).

For Positive and Negative Affect Schedule (PANAS) negative affect (NA), a time × condition effect (*F*_1,41_ = 7.977, *p* = .007, *q*_*rm−anova*_ = .084, η_p_^2^ = 0.163) (Figure 3C) was suggested, but not for PANAS positive affect (*F*_1,41_ = 1.699, *p* = .200, η_p_^2^ = 0.040) (Figure 3D). Post hoc explorations for NA suggested an increase in the WLC group that did not survive FDR correction (*z* = −2.099, *p* = .035, *q*_*wsrt*_ = .07) and no change in the PCT group (*z* = 1.68, *p* = .097, *q*_*wsrt*_ = .097).

#### Automatic Thinking, Cognitive Reactivity, and Emotion Processing

No time × condition interaction was observed for the Leiden Index of Depressive Sensitivity (*F*_1,44_ = 0.001, *p* = .990; η_p_^2^ < 0.001) (Figure 3E), Leuven Adaptation of the Rumination on Sadness Scale (*F*_1,44_ = 0.648, *p* = .425, η_p_^2^ = 0.015) (Figure 3F), Dysfunctional Attitude Scale (*F*_1,39_ = 0.480, *p* = .493, η_p_^2^ = 0.012) (Figure 3G), or Responses to Positive Affect (*F*_1,43_ = 0.636, *p* = .430, η_p_^2^ = 0.015) (Figure 3H).

For Emotion Regulation Questionnaire (ERQ) scores, a time × condition effect for cognitive reappraisal (CR) (*F*_1,43_ = 5.760, *p* = .021, *q*_*rm−anova*_ = .08, η_p_^2^ = 0.118) (Figure 3I) was suggested, but not for emotional suppression (*F*_1,44_ = 0.133, *p* = .717, η_p_^2^ = 0.003) (Figure 3J). Post hoc explorations indicated a nonsignificant increase in CR in the PCT group (*z* = −1.807, *p* = .074) and no change in the WLC group (*z* = 1.299, *p* = .199).

For the Bermond Vorst Alexithymia Questionnaire, no time × condition interaction was observed on the cognitive (*F*_1,43_ = 2.984, *p* = .091, η_p_^2^ = 0.065) (Figure 3K) or affective (*F*_1,44_ = 0.167, *p* = .685, η_p_^2^ = 0.004) subscale (Figure 3I).

### Correlations Between Outcome Variables

**Figure 4:**
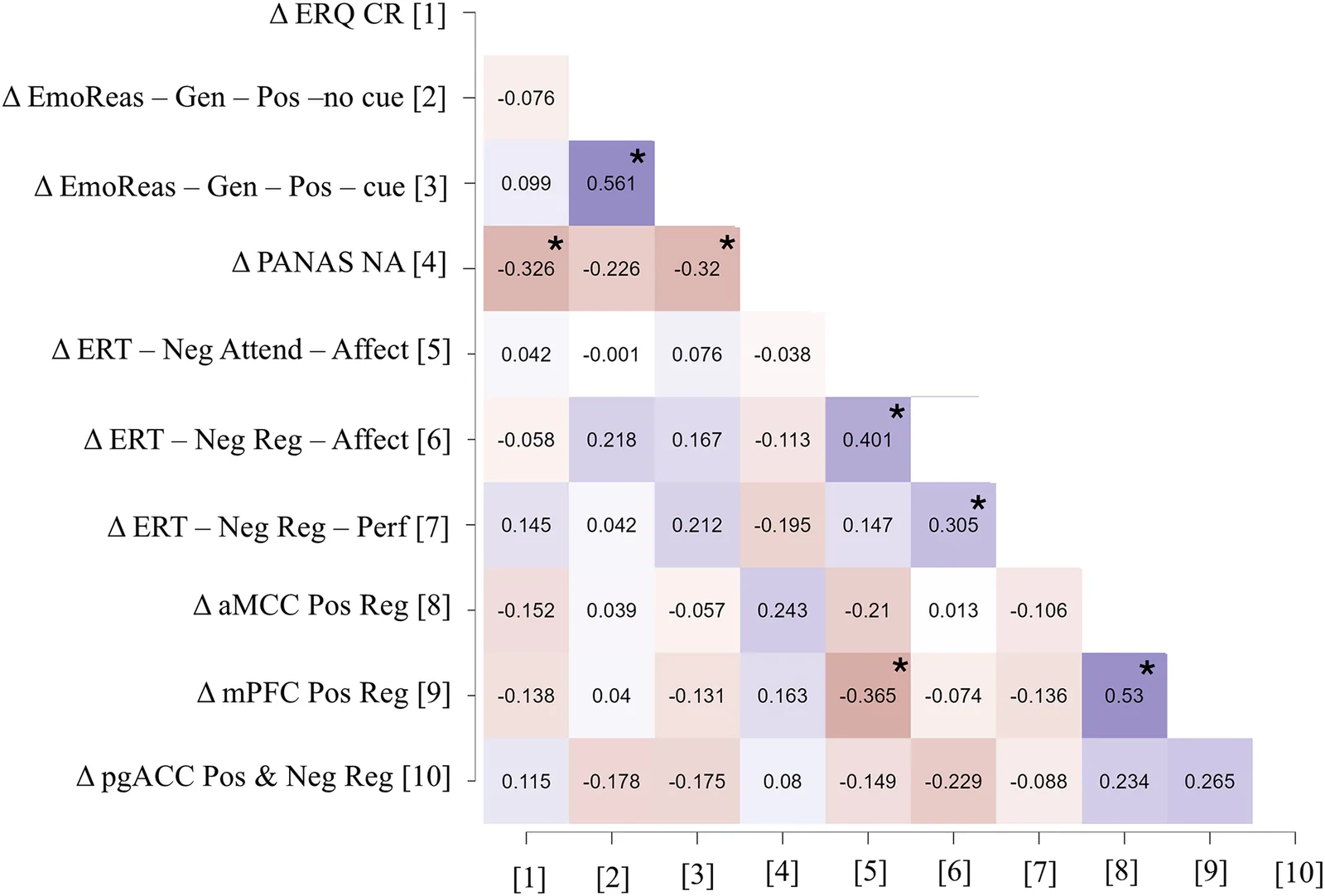
Correlation matrix: relations of change over time. Correlation matrix showing the correlation between change over time in the outcome measures that showed at least suggestive support for an effect of time × condition. Diff, difference (follow-up minus baseline; [Δ]) score for Emotion Regulation Questionnaire Cognitive Reappraisal subscale (ERQ CR [1]); Emotional Reasoning—general cognitions following positive scenarios without an affective cue (EmoReas—Gen—Pos—no cue [2]); Emotional Reasoning—general cognitions following positive scenarios with an affective cue (EmoReas—Gen—Pos—cue [3]); Positive and Negative Affect Schedule—negative affect scale (PANAS NA [4]); Emotion Regulation Task—negative attend condition—affect ratings (ERT—Neg Attend—Affect [5]); Emotion Regulation Task—negative regulate condition—affect ratings (ERT—Neg Reg—Affect [6]); Emotion Regulation Task—negative regulate condition—performance ratings (ERT—Neg Reg—Perf [7]); activation difference in the anterior mid-cingulate cortex between the conditions positive regulate and positive attend (aMCC Pos Reg [8]); activation difference in the medial prefrontal cortex between the conditions positive regulate and positive attend (mPFC Pos Reg [9]); activation difference in the pregenual anterior cingulate cortex between the regulate and attend instructions across negative and positive pictures (pgACC Pos and Neg Reg [10]). The ∗ indicates that the *q* value corresponding to the correlational value survived correction for multiple comparisons.

Finally, we explored correlations (Kendall’s tau) between changes (T1 minus T0) across conditions for primary and secondary outcome parameters that suggested an effect of PCT versus WLC (see Figure 4 for the correlational heatmap). We tested and corrected for 10 variables and 45 tests following the Benjamini-Hochberg procedure (Data S1). Note that the parameters for calculating the correlations were selected post-statistical testing (41); therefore, the *q*-values should be regarded as descriptive and interpreted with care.

Changes in medial prefrontal cortex (PFC) activation during positive upregulation were negatively related to changes in affective ratings following attending to negative stimuli during the task (*r* = −0.365, *q* = .0375) and positively related to changes in dorsal ACC activation during positive upregulation (*r* = 0.530, *q* = .015).

Changes in affective ratings following downregulation of negative emotions was positively related to change in affective ratings after attending to negative pictures (*r* = 0.401, *q* = .0225) and to performance ratings following downregulation of negative emotions (*r* = 0.305, *q* = .0386).

Changes in positive thought reactivity in response to positive scenarios (cue) was negatively related to change in PANAS NA scores (*r* = −0.320, *q* = .0225) and positively related to change in positive thought reactivity in response to positive scenarios (no cue) (*r* = 0.561, *q* = .045).

Finally, changes in PANAS NA scores were negatively correlated with changes in ERQ-CR scores (*r* = −0.326, *q* = .0225).

## Discussion

Here we studied for the first time the neurocognitive mechanisms through which relapse prevention may be facilitated in unmedicated individuals remitted from recurrent depression who are at high risk for relapse. We observed changes in recruitment of medial prefrontal cortical regions during the instructed emotion regulation and strengthening of general self-related positive thinking but no changes in early stages of processing of emotional information. These primary results suggest that PCT modulates the ability and propensity to directly regulate emotional states but not by changing the early processing of emotional information. Furthermore, our results support that targeting regulatory mechanisms that underpin enhanced positive affect and reactivity of positive thinking may be an important target for strengthening resilience against relapse following recurrent MDEs.

Following PCT, less activation of medial prefrontal structures, including the medial PFC, aMCC, and pgACC, was observed during regulation than during attendance compared to the WLC group. For the pgACC, this was observed across downregulation of negative emotions and upregulation of positive emotions, while for the aMCC and medial PFC, the decrease in activation difference between reappraising and attending was only observed for the positive condition. The pgACC has been recognized as a connector hub between brain systems that are implicated in primary emotion processing and effortful control (42, 43, 44) and was found to be hyperactive in the acute depressed state during negative emotion processing based on a large meta-analysis (45). Our results of increasing pgACC over time in the WLC group may reflect symptom worsening and accompanying increased response to emotional information. The medial PFC and aMCC have been linked to effortful regulation (46), attentional control (47), and conflict processing and resolving (48). Together, the results of smaller activation difference over time in medial prefrontal regions during regulation of versus attending to emotional content suggest that less cognitive control resources (49) are needed to either initiate or maintain a regulatory cognitive action, especially for positive emotional states, which are often experienced to a lesser extent following depressive episodes (37). Following similar reasoning, the increase in activation difference in these regions in the WLC group may signal an increased need for regulatory resources with remission with no preventive intervention offered. Contrary to our hypotheses, no changes in engagement and disengagement with shortly presented positive and negative emotional material was observed in the context of an adapted dot-probe task for the PCT versus WLC groups or the IAT.

The changes in medial prefrontal activation following PCT occurred in the context of increased general self-related positive thinking in response to envisioned future situations, less negative affective ratings following the negative blocks during the ERT, and higher performance ratings regarding the ability to implement the regulation instruction. Furthermore, changes in the ability to respond with general self-related positive thinking were suggested to be related to lowered NA in daily life. In contrast to our expectations, we observed no effect of PCT on rumination, cognitive reactivity, severity of dysfunctional attitudes, or depressive implicit self-associations. This lack of effect of PCT on negative thinking converged with earlier studies on PCT (15) and the limited support that was recently found for the cognitive model of depression in explaining treatment effects in patients with acute depression (50, 51, 52) via changing dysfunctional attitudes. However, recent evidence based on network analysis suggests that changes in dysfunctional attitudes are not a direct effect of PCT; however, they are important for lowering the relapse risks and could be obtained by strengthening positive emotions and the ability to implement CR (16). Overall, current evidence suggests that PCT obtains its relapse-preventing effects by targeting regulatory mechanisms that underpin enhanced positive affect and reactivity of positive thinking.

Our results are partially consistent with previous neuroimaging studies that detailed the effects of CBT during the acute depressed phase. No neural effects of CBT were observed in patients with acute depression during an ERT (20,53), but changes during a self-referential processing task were observed in the medial PFC (54), reductions that during regulation of negative autobiographical memories were predictive of positive treatment outcomes (20). In the only other neurocognitive intervention study of remitted depression, medial prefrontal brain regions likewise showed lower activation following mindfulness-based CT in a self versus other blaming task (55). To our knowledge, no other study reported to date has explored effects of CT on the neural correlates of emotion regulation, and none studied effects on positive emotion processing or regulation or included a WL or other control condition. We studied both valence domains, thereby allowing for examining specificity of change. This approach now indicates that the relapse-preventing effects of PCT occur via 2 valence pathways, one via neural efficiency of downregulation of negative emotions, reduced NA responsivity, and increased success of applying CR to regulate negative emotions and another via more neural efficiency of upregulation of positive emotions and increased positive self-related general thinking.

Furthermore, the paths seem to cross; our results indicate that increased positive self-related thinking and less medial prefrontal resources that are needed to regulate positive affect may result in lower NA in response to negative stimuli. This is supported by the explorative correlational analyses that indicate that changes in medial PFC activation during regulation of positive emotions and change in general positive self-related thoughts during the emotional reasoning task were related to lower affective ratings in response to negative stimuli and lower NA in daily life. Thus, PCT may promote the capacity to utilize positive regulation and positive thinking to inhibit NA elicited by daily life stress. However, the current results should be considered suggestive evidence, and future studies should provide validation of these suggested pathways.

These findings align with the compensatory skills hypothesis of CT (56) as well as the retrieval competition account of Brewin (57). The retrieval competition account states that CBT has its effects not by directly changing negative memory representations in memory but instead by changing the relative activation of positive and negative representations. This increases the likelihood that positive memory representations may win the retrieval competition. Previously, positive compensatory skill learning (i.e., positive and constructing way of responding to stressful scenarios) was specifically associated with symptom reduction following CT (58,59) and with lower risk for relapse (60). More recent treatment developments also underline the importance of targeting positive valence for treating depression, anxiety, and suicidality during the acute phase of depression and anxiety, as shown in trials of positive affect treatment (61,62). We now add that PCT delivered during the remitted phase of MDD enhances general positive self-related thoughts and neurocognitive ease to upregulate positive emotions in response to positive experiences, possibly compensating for or buffering against the effects of negative thought/belief activation and risk for subsequent depressive episodes.

This study has several strengths. We provided a careful and rich characterization of clinical, (neuro)cognitive, and affective dimensions of functioning. Also, the study was conducted in a carefully selected sample of adult, unmedicated fully remitted patients who had recovered from at least 2 depressive episodes in the past 5 years and had no other current Axis I or confirmed Axis II psychopathology. Furthermore, remitted participants were randomized to PCT (that was delivered in a well-controlled manner) or a WL condition after completion of the baseline measurement, allowing us to detect changes over time that cannot be attributed to repeated measuring, major reductions in symptom severity, or a bias in motivation during the baseline measurement. We chose to be highly selective to rule out important confounders and maximize treatment efficacy. However, this highly selective sampling also limits the generalizability of findings to the larger population of people with depression who often experience incomplete recovery or report clinically significant symptoms of other psychiatric disorders, including anxiety disorders or alcohol use disorders. Furthermore, sample sizes could be considered small, especially because high-quality fMRI data were not available for all patients for both the baseline and follow-up measurements. Some of our effects nearly reached significance, although medium to large effect sizes were observed that indicated potential clinical relevance. Nevertheless, the small sample might have increased the risk for both inflated estimates of effect sizes and having too low power to detect true effects. The small sample size also prevented us from exploring the effects of age on the treatment mechanisms. Another limitation of this study is the lack of a control condition in which patients were offered some other form of active treatment. This limits our ability to draw conclusions about the specificity of effects attributable to PCT. Finally, the higher dropout in the PCT group might have biased results of the final included sample toward higher symptomatic stability (because patients who dropped out reported experiencing an increase in symptoms) or motivation to prevent relapse.

### Conclusions

Results from the current mechanistic study suggest that a number of cognitive-affective changes could explain the demonstrated prophylactic effect of PCT. Compared to a WLC condition, PCT decreased the medial prefrontal regulatory resources needed to enhance (especially positive) affect using CR techniques, increased reactivity of positive self-related thinking, and lowered reactivity of NA. Early emotional attentional biases and negative cognitive reactivity seem less relevant in understanding preventive effects. These results support the notion that strengthening and shifting cognition and affect to more positive content may protect against the activation of negative cognitions and affect in the face of stress and negative life events.

## Supplementary Material

Supplemental Sections 1–11

Benjamini-Hochberg Corrections

Key Resources Table
