# Supplementary material for: Neurocognitive Mechanisms of Preventive Cognitive Therapy as Relapse Prevention in Remitted Major Depressive Disorder: A Randomized Controlled Trial
Source: Biol Psychiatry Glob Open Sci. 2026 Jun 25;6(6):100781. doi: 10.1016/j.bpsgos.2026.100781 (PMC13527494; doi:10.1016/j.bpsgos.2026.100781)
Supplement: Supplementary file 1 [file mmc1.pdf]

SUPPLEMENTARY INFORMATION

Neurocognitive Mechanisms of Preventive Cognitive Therapy as Relapse Prevention in Remitted Major Depressive Disorder: A Randomized Controlled Trial

van Tol *et al.*

SUPPLEMENT 1: FLOW CHARTS 3

SUPPLEMENT 2: TREATMENT INTEGRITY, PROTOCOL ADHERENCE AND HELPING ALLIANCE ... 6

SUPPLEMENT 3: OVERVIEW OF MEASURES PER DOMAIN 7

SUPPLEMENT 4: INFORMATION ABOUT THE EMOTION REGULATION TASK........ 10

SUPPLEMENT 5: EXTRA INFORMATION ABOUT THE ARDPEI TASK........ 12

SUPPLEMENT 6: EXTRA INFORMATION ABOUT THE IMPLICIT ASSOCIATION TASK........ 14

SUPPLEMENT 7: EXTRA INFORMATION ABOUT THE EMOTIONAL REASONING TASK 15

SUPPLEMENT 8: FMRI PREPROCESSING AND MODELLING DETAILS 17

SUPPLEMENT 9: COMPOSITE REGION OF INTEREST MASK 19

SUPPLEMENT 10: DESCRIPTIVES OF OUTCOME MEASURES FOR THE PCT AND WLC GROUPS 20

SUPPLEMENT 11 MAIN TASK EFFECTS OF THE EMOTION REGULATION TASK 23

SUPPLEMENT REFERENCES........ 24

Supplement 1: Flow Charts

The Neurocognitive Working Mechanisms of the Prevention of Relapse In Depression (NEWPRIDE) study was set up to achieve several aims.

Aim 1) study neurocognitive factors underpinning the vulnerability for relapse;

Aim 2) understand the neurocognitive working mechanisms of PCT.

Aim 3) predict longitudinal treatment effects based on pre-treatment neurocognitive characteristics.

Aim 4) validate the pupil dilation response as a marker for prefrontal activity, reflecting emotion regulation capacity and therapy success.

For **Aim 1**, the first 50 individuals with rrMDD (recurrent MDD, but currently in remission) included in the study were compared to 25 never-depressed controls to study residual abnormalities in neurocognitive processes in people who experienced multiple depressive episodes in the recent past. Never-depressed individuals only participated in the baseline measurement. See van Kleef et al. for the report of results related to emotion regulation (1).

For **Aim 2**, the first 50 rrMDD individuals included in the study (the people included for aim 1) were following the baseline measurement randomized to receive either PCT or to a waiting list condition.

For **Aim 3**, an additional 25 rrMDD individuals were included for the full study protocol, though during randomization, only receiving PCT was an option. rrMDD individuals did not now about this and were informed to be randomized to either PCT or the waiting list condition. For these additional 25 rrMDD individuals, another MRI-scanner had to be used, due to unforeseen change of scanners at the time of study planning.

The current report focuses on Aim 2 and reports on the immediate effects of PCT in comparison to a waiting list condition (See Figure S2; right figure)

When rrMDD individuals were randomized to one of the conditions, but dropped out of the study, they were replaced as much as possible, given the focus of this study on the effects of PCT when delivered. In The CONSORT FLOW DIAGRAM (See Supplemental Figure 1.1), the flow of all rrMDD individuals is depicted.

Supplemental Figure 1.2 depicts the study phases and highlights of which groups and measurements data was included for the current report. A full description of the protocol is provided in the protocol paper (2).

Supplemental Figure S1.1: CONSORT 2010 Flow Diagram – Full NEWPRIDE trial

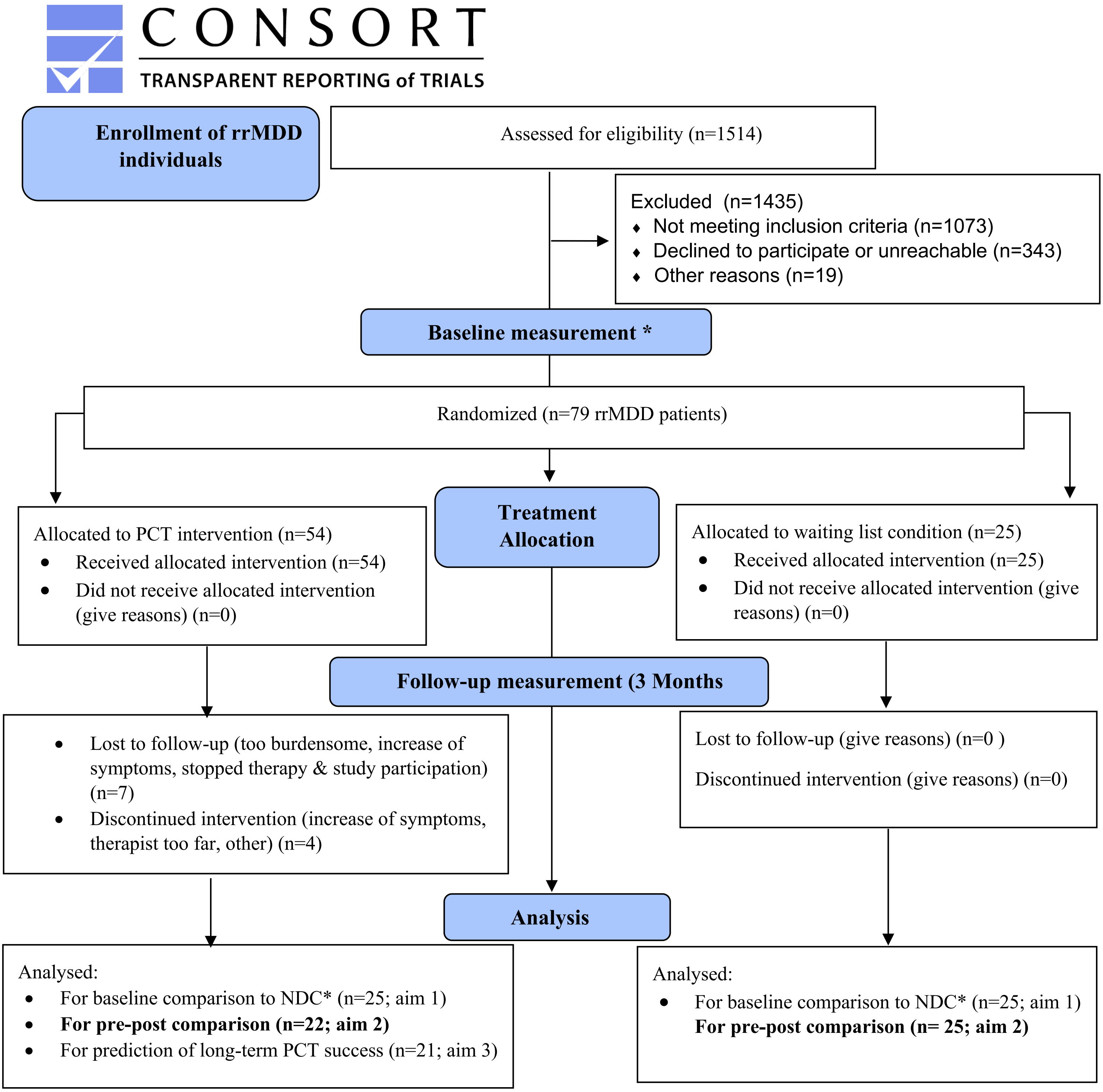

* For the baseline measurement, 95 never-depressed controls (NDC) participants were screened for eligibility, of which 25 were included. Reasons for exclusion were: presence of current or past psycho-pathology (n=10), non-MRI compatibility (n=7), visual impairments (n=6), non-native Dutch (n=3), declined to participate/no contact (n=17), too old (n=5), no match to rrMDD group (n=10), too high depressive symptomatology as measured with the Inventory of Depressive Symptomatology (n=2), not included because target of n=25 was met (n=10).

Supplemental Figure S1.2: NEWPRIDE Flow chart – Randomized Controlled Trial of Preventive Cognitive Therapy vs. Waiting List
Flow chart describing the study design of the Neurocognitive Working Mechanisms of Preventing Relapse in Depression (NEWPRIDE), focusing on the full study design, including 3 aims (left) and the here presented primary analyses related to aim 2 (right). Full study design has been described in van Kleef et al. (2019).

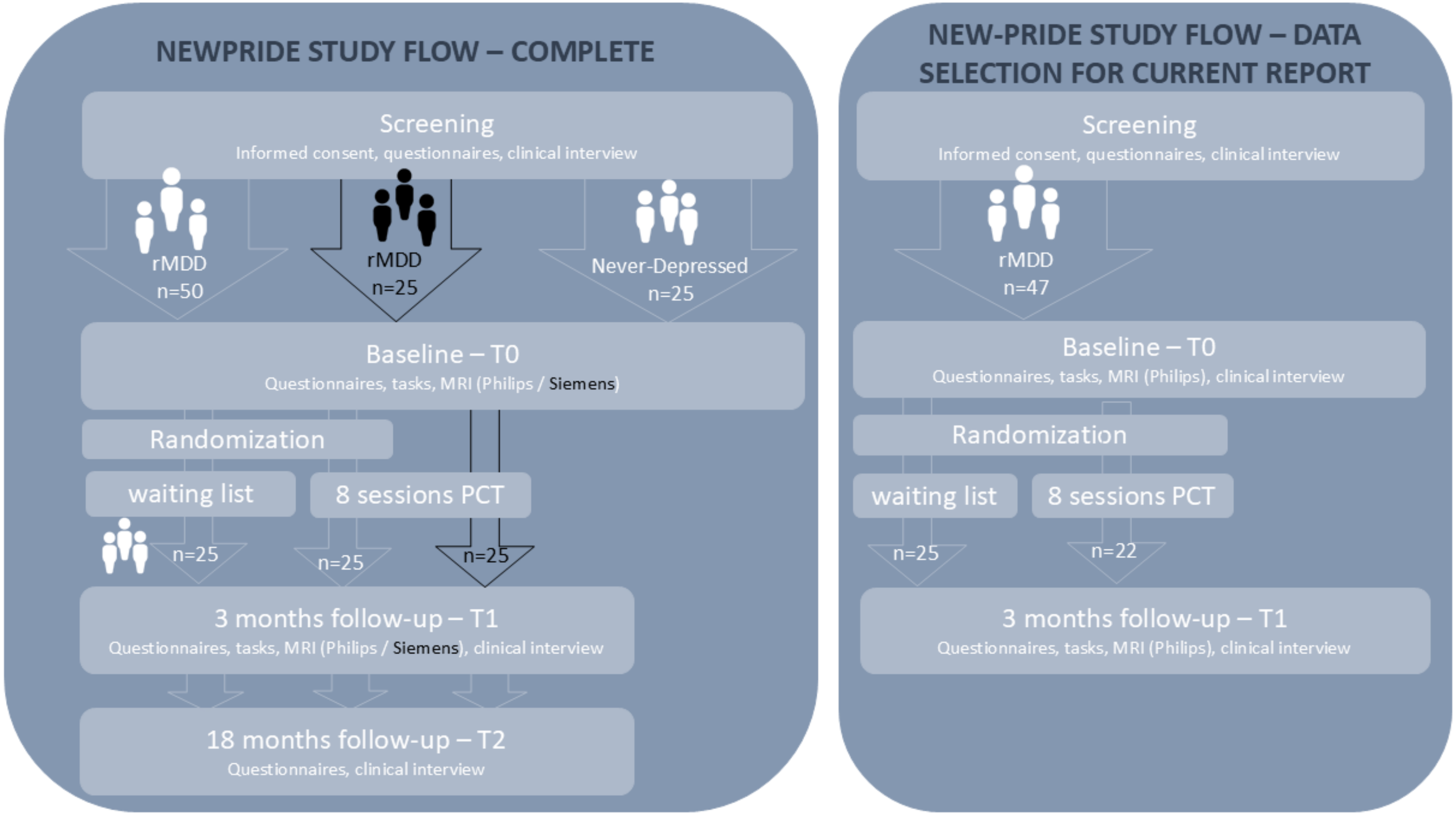

Supplement 2: treatment integrity, protocol adherence and helping alliance

To ensure adequate treatment integrity, therapists were instructed to strictly follow the treatment manual (3,4) and were supervised by a licensed cognitive therapy supervisor (EvV). Prior to delivering PCT in context of the RCT, all therapist delivered the full PCT to one remitted MDD-individual (who did not participate in the RCT) under supervision of EvV in order to ensure sufficient knowledge and experience with delivering the treatment.

Protocol adherence was assessed by reviewing each therapy session by two independent researchers, who scored presence of predefined therapy 'ingredients'. A score of 100 represented perfect adherence to protocol, a score of 0 represented none of the aspects were present. After scoring adherence independently, the two researchers discussed their scores and reached a consensus score for each individual session.

After the treatment, the quality of the therapeutic alliance was rated by both the therapist and the participant after finalizing the treatment using the Helping Alliance Questionnaire (5), consisting of 19 statements related to the therapeutic alliance that were rated on a scale from 1 (strongly disagree) to 6 (strongly agree). A total score was calculated.

Mean protocol adherence scores and HAQ scores rated by Patients and Therapists are provided in Figure S2.1.

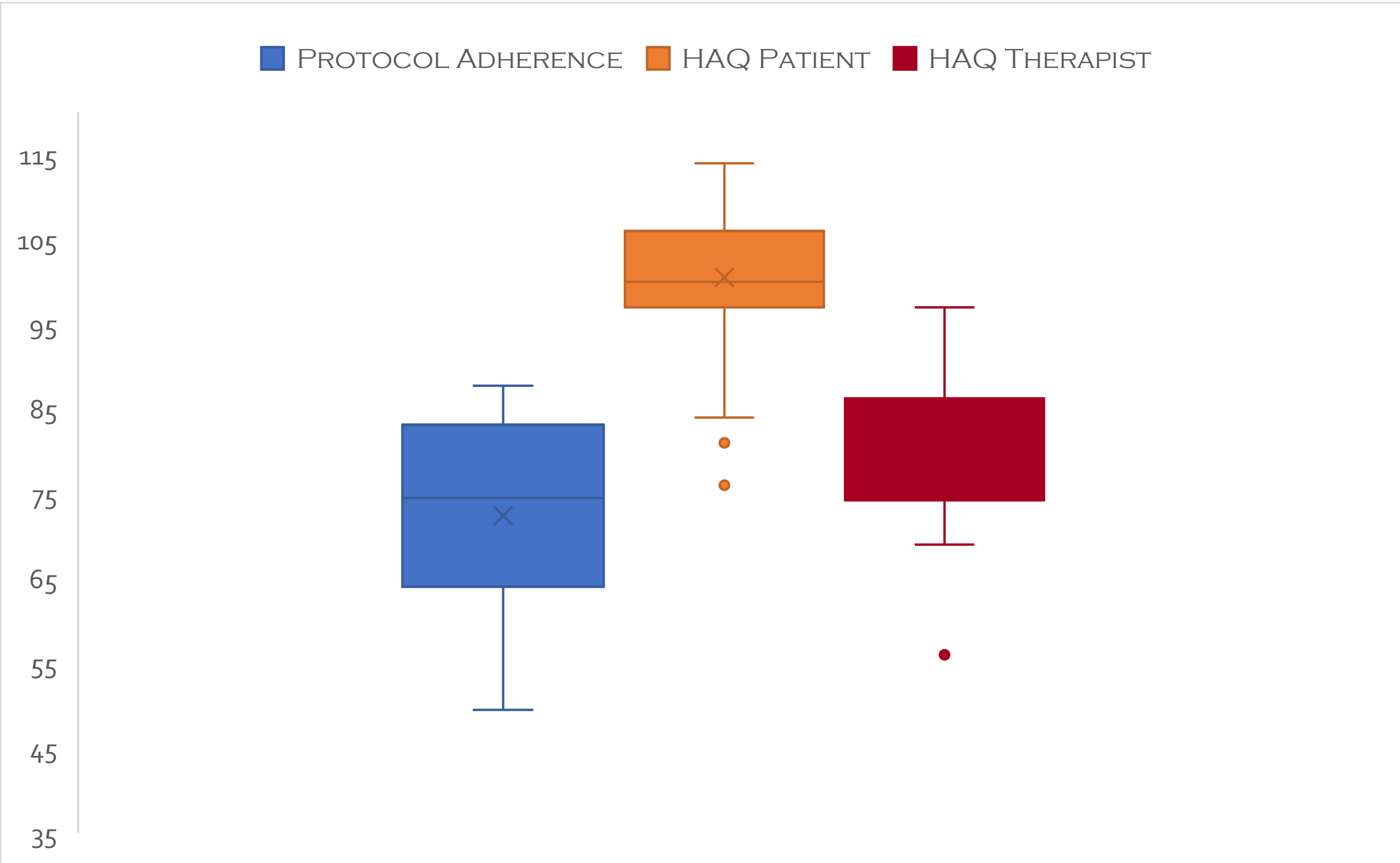

Figure S2.1. Boxplots depicting the Protocol Adherence (blue; max possible score: 100) and the Helping questionnaire scores as reported by the patients (orange) and the therapists (red) in the PCT-condition.

Supplement 3: Overview of measures per domain

Depressive symptomatology

At baseline and follow-up, patients were carefully characterized with respect to their depressive symptomatology (Inventory of Depressive Symptomatology, [IDS-SR](6,7)) experienced in the past week, consummatory pleasure experienced in the past two weeks (Domains of Pleasure Scale, [DOPS](8,9)), and characteristics of positive and negative affective states in the past week on a VAS scale ranging from zero till 100 (Positive and Negative Affect Scale, [PANAS](10,11)). We added two questions to the PANAS and asked participants to rate the experienced variability and stability of their positive and negative affect in the past week on a VAS scale ranging from zero till 100.

At three-month follow-up, the SCID-I module (12) for current and past MDEs was repeated (this was also administered during the screening phase), and the SCID-I Life Chart Method (13) was used to assess presence and burden of depressive symptomatology per month since the baseline measurement.

Automatic thinking patterns and cognitive reactivity style

Cognitive response profiles were assessed with the Leiden Index of Depression Sensitivity, 2nd Revision [LEIDS-RR](14), providing a measure of the following cognitive reactivity styles: Hopelessness, Rumination, Aggression, Acceptance, and Control. Ruminative tendencies were assessed using the Leuven Adaptation of the Rumination on Sadness Scale [LARSS](15,16), which provides characteristics of Causal Analysis, Understanding and Uncontrollability of ruminative thinking. Rumination on positive thoughts and emotions was measured using the Responses to Positive Affect questionnaire [RPA](17), which provide characteristics related to Self-focused, Dampening and Emotion-focused styles of processing positive thoughts and emotions.

Strength of latent dysfunctional attitudes was assessed with the Dysfunctional Attitude Scale (DAS-A)(18). Reactivity of automatic negative and positive thoughts was assessed with the Emotional Reasoning Task (19), a computerized task in which participants rated the likelihood of certain specific and general self-related thoughts arising in response to twelve positive and twelve negative scenarios in which the patient had to imagine themselves. Of these scenarios, half were followed by a description (cue) of how the situation would make them feel (i.e. 'you feel happy' or 'you feel sad'). Since these scenarios were processed on a conscious level, two task versions were created including different (but matched with regard to content and valence) scenarios. Half of the participants were randomized to version A for the baseline measurement, and the other half to version B. For the follow-up measurement, task versions were switched around. See Supplement 7 for more details.

Affective reactivity and emotion regulation capacity and strategy use

We derived information on affective responsivity and success of applying cognitive reappraisal when confronted with emotional material from the Emotion Regulation Task (ERT), which was performed during functional Magnetic Resonance Imaging (fMRI) (See supplement 4 for full details)(20). The ERT was designed to measure regional brain activity during passive attending to negatively, positively and neutrally valenced pictures, and active regulation of negatively and positively valenced pictures. The task consisted of 15 pseudorandomized blocks, with three repetitions per condition. Task conditions (five in total) included: attending to neutral pictures, attending to negative pictures, attending to positive pictures, downregulation of negative pictures, and upregulation of positive pictures. In each block, four pictures were displayed for 8 seconds each, with a jittered interval varying between 450 and 1250 msec. Between conditions, a fixation cross was shown (with jittered duration between eight and 14.5 seconds).

Pictures were carefully selected from the IAPS set (International Affective Pictures System; (21)) and matched for the positive and negative conditions (i.e. attending and regulation) on valence, arousal, dominance and visual scenery. Since pictures were consciously processed, we created different task versions for the baseline and follow-up measurements. Half of the participants was randomized to version A at baseline and the other half of the participants to version B, and task versions were switched for the follow-up. Version A and B included the same pictures, but they were differently allocated to attend and regulation conditions.

During the attend condition, participants were instructed to simply watch the picture and to naturally experience any emotion that may arise. During the regulation condition, participants were instructed to apply cognitive reappraisal techniques to alter the intensity of elicited emotions. For the negative conditions, they were instructed to apply cognitive reappraisal techniques to reduce the intensity of the experienced negative emotions. For the positive conditions they were instructed to apply cognitive reappraisal techniques to increase the intensity of experienced positive emotions. The regulation techniques were trained prior to scanning until satisfactory. More details on the ERT can be found in Supplement 4.

Affective responsivity to emotional stimuli was measured by asking patients how they felt following each block of four pictures. Patients rated their affect on a VAS scale ranging from zero [very sad] to 100 [very happy]. Success of applying cognitive reappraisal was assessed by asking patients after each regulation block how well they could execute the task instruction of applying cognitive reappraisal. Patients rated their performance on a VAS scale ranging from zero [not at all] to 100 [very well].

Daily use of emotion regulation strategies (i.e. Cognitive Reappraisal and Emotional Suppression) was assessed with the Emotion Regulation Questionnaire (ERQ)(22).

We also assessed affective and cognitive dimensions of alexithymia using the Bermond-Vorst Alexithymia Questionnaire [BVAQ](23). Alexithymia (literally meaning 'no name for feelings') is a personality trait that is highly relevant for adequate emotion regulation. The cognitive dimension reflects how well an individual is able to identify, verbalize and analyze feelings, whereas the affective dimension reflects the extent to which an individuals is affected by emotional stimuli and how well someone is able to fantasize.

Cognitive-affective biases

To measure attentional biases towards and away from negative and positive information, respectively, we employed an adapted version of the Attentional Response to Distal vs. Proximal Emotional Information (ARDPEI) task (24). This task is an adaptation of the classical dot-probe task that allows differentiation between engagement and disengagement biases. Originally, the task was designed to measure attentional biases related to anxiety and included only trials featuring negative and neutral pictures, which were presented for 100 msec in half of the trials and 500 msec in the other half of the trials (i.e. 32 trials per valence per duration). Given the relevance of positive attentional biases, we added 64 trials including positive pictures. Also, because biased attention has been suggested to occur at a later processing stage in depression than in anxiety disorders, we presented pictures for 500 msec and 1000 msec. Details on the task design, picture selection, and calculation of the bias indices can be found in Supplement 5. Because our hypotheses are directed at changes in emotional aspects of biased processing, we analysed the negative engagement and disengagement indices and positive engagement and disengagement indices separately.

A computerized Implicit Association Task (IAT;(25)) was administered to assess the strength of implicit negative self-associations participants may have regarding themselves. The IAT is a word-sorting task for which individuals have to indicate as fast as possible the membership of certain words

to two categories: the first related to oneself or others and the second to the emotional loading (negative/positive). The two categories appeared in either the left or right corner of the computer screen and a sequence of words related to these categories appeared in the middle of the screen (See Supplement 7 for stimuli and block sequence). The IAT measures whether one has more automatic self-associations with positive or negative attributes based on response latencies. The relative strength of each participant's self-associations was indexed by calculating a DnoEP-score (the standardized score used for the calculation of within-person differences based on correct trials only, in line with (25)). Higher IAT-scores indicate stronger negative self-associations.

Supplement 4: Information about the Emotion Regulation Task

Task design

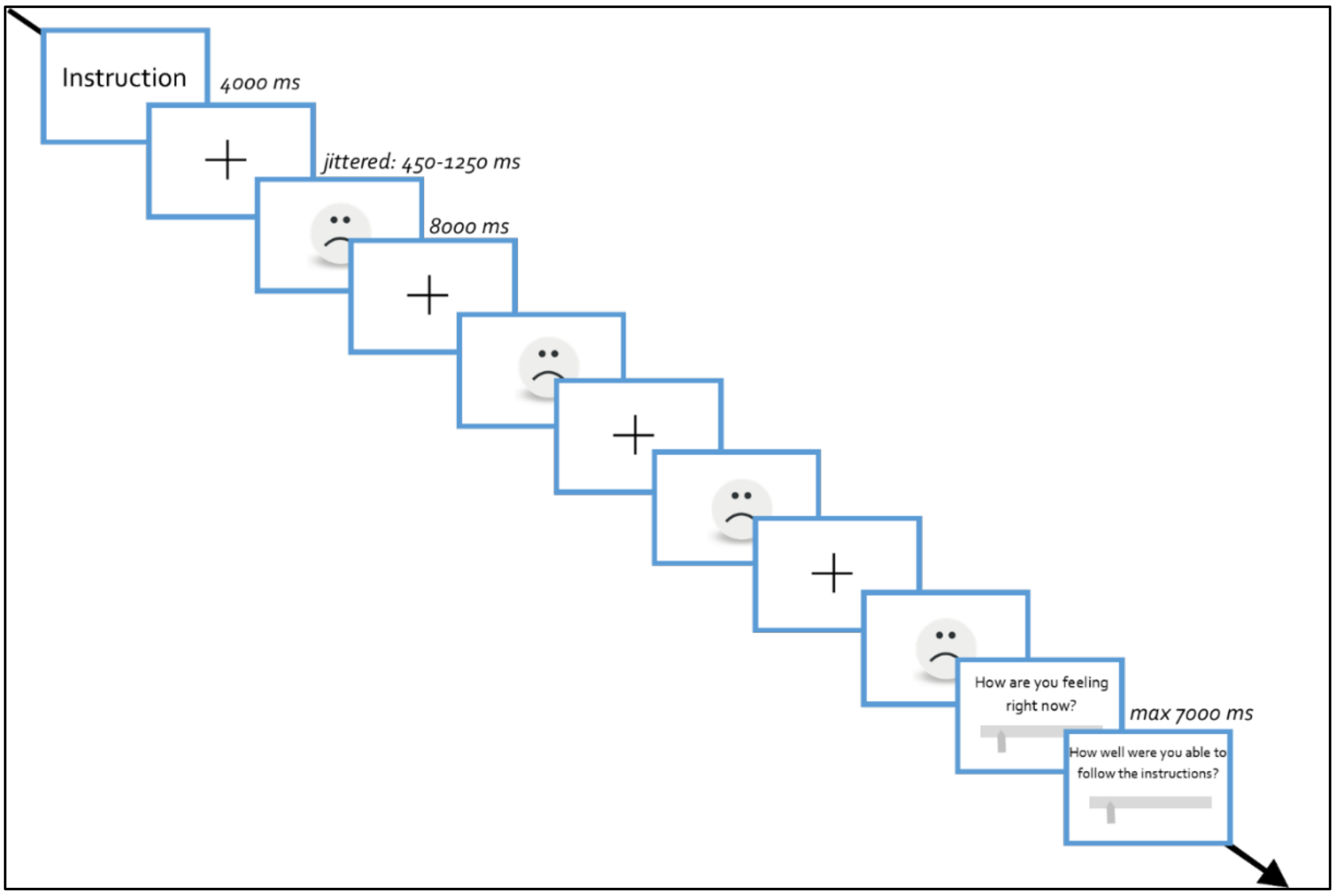

Figure S4.1. Emotion Regulation Task design: order of presentation during one block; example of one experimental block. The instruction ("attend" or "regulate") is presented, followed by four IAPS-pictures of the same valence, and two VAS ratings. The instruction and pictures were separated with fixation jitters lasting between 450-120ms, consisting of a "+". In total, one block took approximately 50 seconds and each block was separated by a jittered fixating, varying from 8 to 14.5 seconds.

IAPS pictures

Pictures in the negative category contained both sad and fearful images. Within each category, pairs were created with pictures containing similar content, so pictures for attend- and regulate conditions could easily be matched. The pairs for the two conditions were matched on valence, arousal and dominance (see Table S4.1). Ratings for valence, arousal and dominance were derived from Mikel et al. (26) and Lang et al.(21).

Table S4.1

**Category**	**Pair**	**Nr of pairs**	M(SD)<br>**Valence**	**Arousal**	**Dominance**
Negative					
Sad	Pair 1	6	2.04 (1.39)	5.32 (2.15)	3.43 (2.01)
	Pair 2	6	2.19 (1.44)	5.29 (2.21)	3.55 (2.12)
Fearful	Pair 1	6	2.05 (1.39)	6.78 (2.01)	2.95 (2.08)
	Pair 2	6	2.10 (1.39)	6.79 (2.07)	2.84 (2.07)
Positive	Pair 1	12	7.5 (1.38)	5.25 (2.11)	6.13 (1.87)
	Pair 2	12	7.54 (1.40)	5.18 (2.18)	6.15 (2.01)
Neutral	Pair 1	12	5.30 (1.23)	3.20 (1.97)	0.79 (3.29)
	Pair 2	12	5.23 (1.30)	3.32 (1.97)	0.82 (3.29)

Table S4.1. Scores on valence, arousal, dominance and luminance of pictures selected from the IAPS for the ERT.

Supplement 5: Extra information about the ARDPEI task

We employed an adapted version of the Attentional Response to Distal vs. Proximal Emotional Information (ARDPEI) task (38), an adaptation of the classical dot-probe task that allows differentiation between engagement and disengagement biases to briefly (500 msec and 1000 msec) presented visual stimuli (see (37)). We analysed the engagement and disengagement index-parts for positive and negative conditions separately.

Bias calculation

Biases were calculated as follows:

For the engagement bias index: anchor probe distal to emotional image: RT for target probe distal to emotional image – RT for target probe proximal to emotional image (see Figure S4).

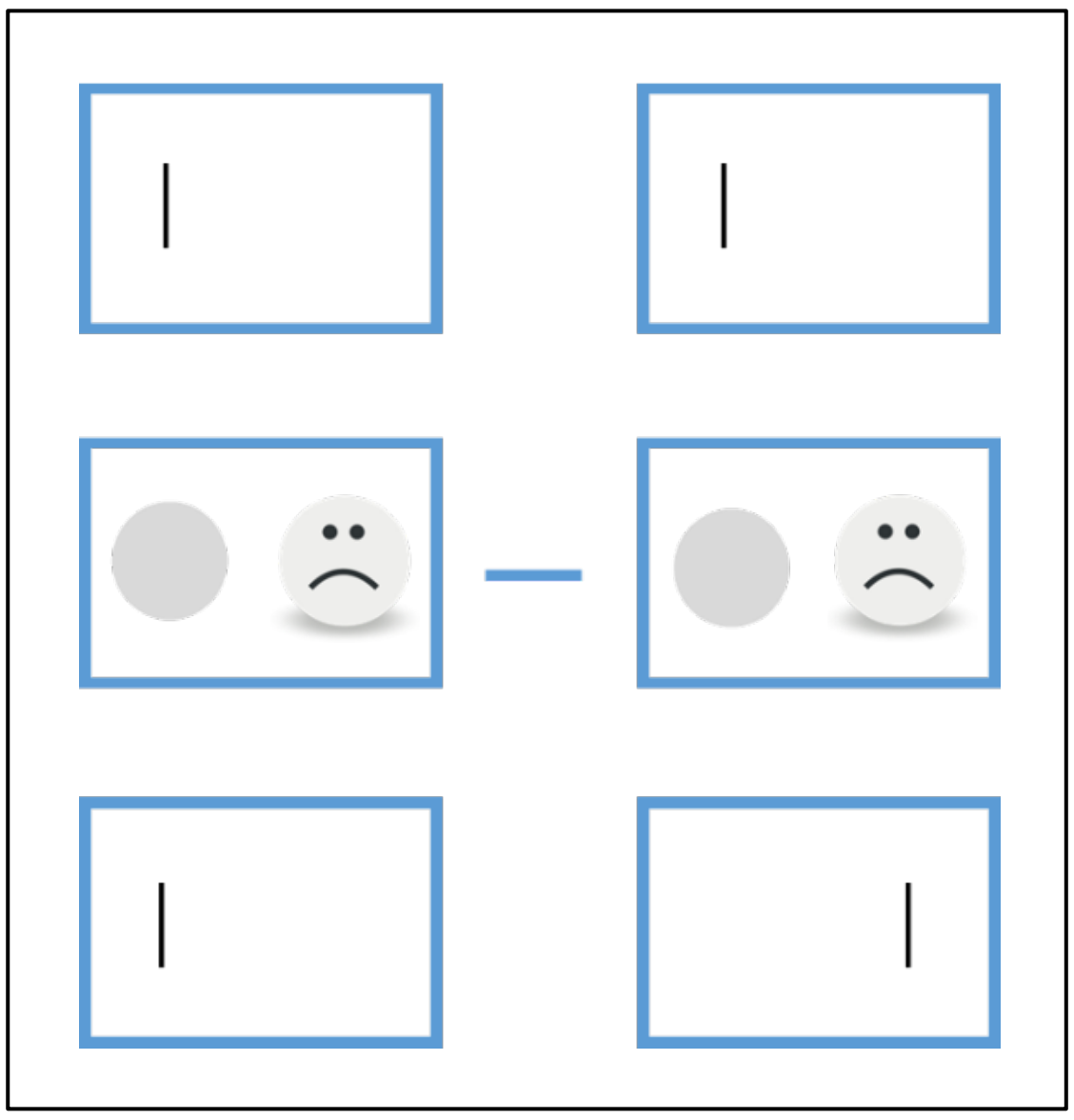

Figure S5.1: calculation of the engagement bias index

For the disengagement bias index: anchor probe proximal to emotional image: RT for target probe distal to emotional image – RT for target probe proximal to emotional image (see Figure S5).

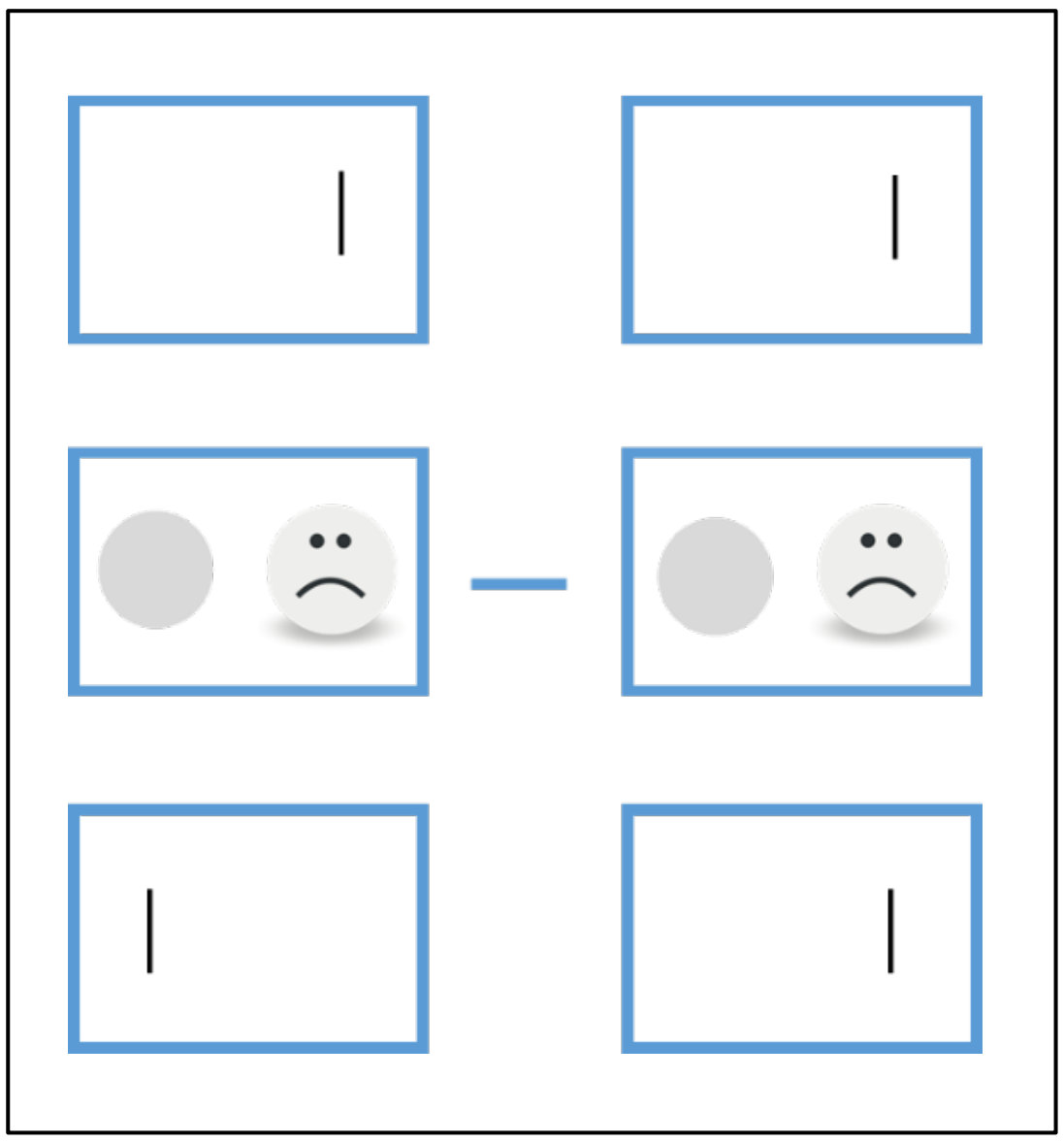

Figure S5.2. calculation of the disengagement bias index

IAPS pictures

Each category contained 32 pictures. We ensured that pictures were not similar to the pictures selected for the Emotion Regulation Task. Pictures for distal and proximal conditions within each valence category were matched for valence, arousal, dominance and luminance (see Table S)5.1. Ratings for valence, arousal and dominance were derived from Mikel et al. (26) and Lang et al. (21).

Table S5.1

		M(SD)			
		**Valence**	**Arousal**	**Dominance**	**Luminance**
Negative	Distal	2.39 (.42)	5.51 (.76)	3.71 (.52)	110.23 (32.43)
	Proximal	2.27 (.35)	5.39 (.56)	3.65 (.47)	109.13 (28.48)
Positive	Distal	7.67 (.29)	5.24 (.90)	6.02 (.66)	116.10 (33.19)
	Proximal	7.74 (.29)	5.17 (.83)	6.13 (.56)	116.86 (36.46)
Neutral	Distal	5.06 (.36)	3.60 (.65)	5.59 (.48)	105.53 (35.58)
	Proximal	5.11 (.40)	3.45 (.61)	5.59 (.43)	101.93 (35.05)

Table S.5.1 Scores on valence, arousal, dominance and luminance of pictures selected from the IAPS for the ARDPEI task.

Supplement 6: Extra information about the Implicit Association Task

A computerized Implicit Association Task (IAT;((25)) was administered to assess the strength of implicit negative self-associations participants may have regarding themselves. The IAT measures whether one has more automatic self-associations with positive or negative attributes based on response latencies, indexed by calculating a DnoEP-score (based on correct trials only, in line with (25)). Higher IAT-scores indicate stronger negative self-associations.

<u>Stimuli</u>

In our version of the IAT, the target labels were 'me' and 'others', and the attribute labels were 'negative', and 'positive'.

The stimuli belonging to the target labels were 'I', 'myself', 'self', 'my' and 'own' for label 'me', and 'other', 'you', 'they', 'them', 'themselves' for label 'others'.

The stimuli belonging to the attribute labels were 'useless', 'pessimistic', 'inadequate', 'negative', and 'meaningless' for label 'negative, and 'positive', 'optimistic', 'valuable, 'cheerful', and 'active' for label 'positive', similar to earlier studies (27–29).

<u>Example</u>

Figure S6 shows an example. The attribute stimulus presented in the middle (in this case 'worthless') has to be categorized into one of the two target labels (in this case 'negative' or 'positive') by pressing a left or right key. Faster response times during categorizing when 'negative' is coupled to 'self' are thought to reflect higher depressed self-associations.

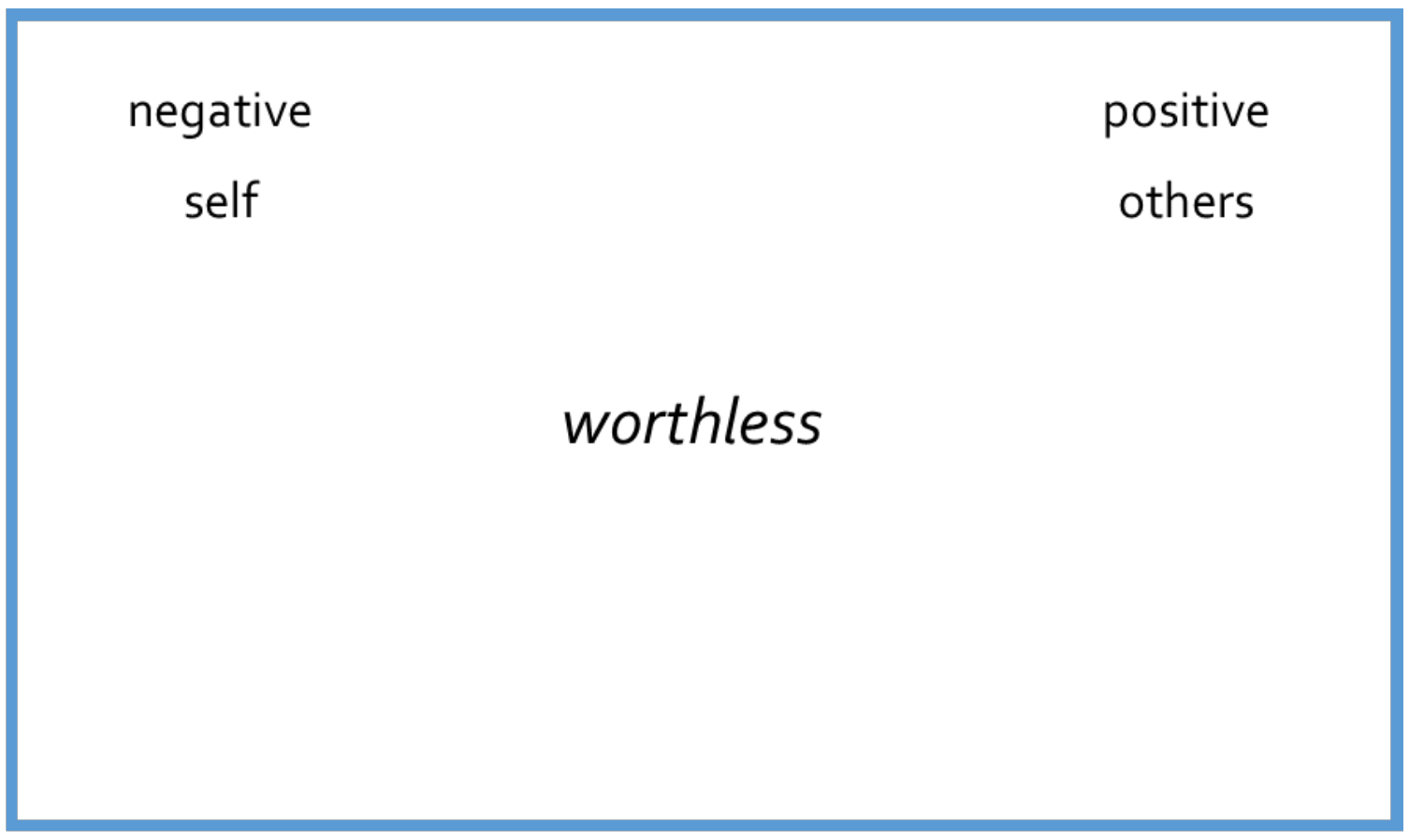

Figure S6.1. Example of computer screen presented to the participants during the IAT.

Supplement 7: Extra information about the Emotional Reasoning Task

The Emotional Reasoning Task is an adapted version of the task by Arntz et al.(19), originally designed for assessing emotional reactivity to cognitions in anxiety disorder. The task has been used in many studies since, in various populations (30–32).

The Emotional Reasoning Task is a computerized task in which participants rate the likelihood of certain specific and general self-related thoughts arising in response to twelve positive and twelve negative scenarios in which the participant had to imagine themselves. Of these scenarios, half were followed by a description (Cue) of how the situation would make you feel (i.e., 'you feel happy' or 'you feel sad').

For this study, we created 24 mildly negative and 24 mildly positive stories in Dutch. See below for an example of a negative and a positive story in English. Stories were created for three categories per valence, with two variations in topic per category. Each task version (A and B) included two stories per variation. See Table S7.1 for the categories and variation in topic. Stories can be requested from the corresponding author.

**Negative**		
'I can't do anything right'		
	Clumsiness in social situations	2 x for version A
		2 x for version B
	Clumsiness in individual situations	2 x for version A
		2 x for version B
Being pessimistic about the future		
	Delivering	2 x for version A
		2 x for version B
	Expecting bad news	2 x for version A
		2 x for version B
Being worthless		
	Social rejection	2 x for version A
		2 x for version B
	Forgetting something / making a mistake	2 x for version A
		2 x for version B
**Positive**		
'I'm capable to do a lot'		
	Skilled in social situations	2 x for version A
		2 x for version B
	Skilled in individual situations	2 x for version A
		2 x for version B
Being optimistic about the future		
	Delivering	2 x for version A
		2 x for version B
	Expecting good news	2 x for version A
		2 x for version B
Being valuable		
	Belonging	2 x for version A
		2 x for version B
	Remembering things	2 x for version A
		2 x for version B

Table S7.1. Categories for which stories were created

During the process of designing the stories, they were rated by four psychologists on how the stories would make them feel about themselves, so adjustments could be made so that negative and positive stories elicited similar levels of emotion, and the version used at baseline and the one at follow-up (A or B) could be matched with regard to level of emotion. Raters were asked: "Read the story, and imagine you are in this situation. How negative or positive would you feel about yourself?" Answers were scored on a scale from -2 (very negative) to 2 (very positive) with 0 being neutral. See Table S7.2 for mean scores of the final version of the stories.

	Mean valence	Standard deviation
Set A negative	-0.58	0.64
Set B negative	-0.73	0.73
Set A positive	1.23	0.55
Set B positive	1.42	0.70

Table S7.2. Mean and standard deviation of the raters scores on the negative and positive stories per task version

<u>Task design and example</u>

The stories were pseudorandomly presented to the participants on a computer screen. Participants were shown twelve negative stories and twelve positive stories, of which half were followed by a cue of how the situation would make you feel ('You feel sad' or 'You feel happy'). All stories were followed by a general and a specific statement about oneself, and participants were asked to rate the extent to which they believed these statements were true on a VAS scale from 0 to 100.

Examples of scenarios of the Emotional Reasoning Task

Example of a negative story: "'*You cycled to the station to take a train. There you see that the bicycle parking is already quite full. When you've found a spot and parked your bike, you knock over the bike next to yours. You put it straight up. [you feel sad].*

Specific cognition: *"You think: "I'm a very clumsy person".*

General cognition: "*You think: I can't do anything right".*

Example of a positive story: "*You haven't spoken to one of your friends in quite a while and proposed to do something nice together. You've made an appointment to meet in a pub. When you enter the pub, you see that your friend has already arrived. [You feel happy]"*

Specific cognition: *"You think: I'm a good friend".*

General cognition: *"You think: I'm valuable".*

Supplement 8: fMRI preprocessing and modelling details

Image acquisition

Scanning was performed on a 3 Tesla Philips Intera scanner (Philips, Best, the Netherlands), located at the Cognitive Neuroscience Center of the University Medical Center of Groningen. During fMR-imaging, patients performed the Emotion Regulation Task (see above), which had a duration of 16 minutes. Functional images were acquired using a T2*-gradient echo-planar sequence with the following parameters: TR=2000 ms, TE=30 ms, FOV=224 x 129.5 mm, image matrix=64 x 62, and voxel size=3.5 x 3.5 x 3.5 mm. Thirty-seven descending axial slices were acquired per volume to achieve whole-brain coverage. For morphometry and registration purposes, a T1-weighted structural scan (170 slices, TR=9 ms, TE=3.53 ms, FOV=256 x 256 mm, image matrix=256 x 256, voxel size=2 x 2 x 2 mm) was acquired.

In the same session a verbal working memory task and scanning during uninstructed rest ('resting state') was performed, in addition to arterial spin labeling, results of which will be reported elsewhere.

Preprocessing and first-level modelling

Functional MRI data was pre-processed and analysed on the subject-level per session (baseline and follow-up, separately) with statistical parametric mapping software (SPM12 v7487; Welcome Trust Centre for Neuroimaging, London, UK), implemented in Matlab 8.5.0 (R2015a; Mathworks, Natick, MA, USA). Scans were manually reoriented to the anterior-posterior commissure plane, realigned, co-registered (anatomical to functional plane using session specific anatomical scan), normalised to MNI space, and spatially smoothed (full-width half-maximum Gaussian kernel of 8mm). Furthermore, framewise displacement was calculated using a threshold of 0.9 mm (33), and volumes containing high slice-to-slice intensity-value differences (related to motion and scanner artefacts) were identified to assess data quality and to be censored in first-level models. If head movement exceeded 3mm (a voxels-width) in any direction, participants' data were excluded from the final analysis.

The task data was modelled in an event-related fashion at subject-level including regressors for the five task conditions (Attend Neutral, Attend Negative, Attend Positive, Regulate Negative, Regulate Positive) where onset and duration of each picture was modelled. Additionally, first-level models included regressors modelling instructions, rating periods, stimulus presentation and their time and dispersion artefacts, and volumes marked as motion or artefact, which were convolved with a canonical HRF. Due to long between-block intervals within conditions, the high-pass filter was set to 0.0052 Hz (192 s).

After model estimation, the following contrast images were calculated, reflecting activation differences between conditions: [Negative Down-Regulate > Negative Attend] and [Positive Upregulation > Positive Attend].

Linear Mixed Effects Modelling

To test for Time x Treatment Condition effects on the contrast maps reflecting regulation of positive and negative images (vs. attendance), linear mixed effects models (LME) were set-up using the AFNI (34) 3dLME tools (35). The linear-mixed effects approach was chosen as it allows to deal with randomly missing data for either baseline or follow-up scan owing to technical problems. We modelled both fixed and random effects, taking the non-independence of data points into consideration.

We set up three LMEs to test for Time x Condition effects on regulation (>attend) related activation. The first LME included Condition (two levels: PCT, WLC) as between-subjects fixed factor and Time (two levels: baseline, follow-up) and Valence (two levels: negative, positive) as within-subjects factors. The contrast images [Negative Down-Regulate > Negative Attend] and [Positive Upregulation > Positive Attend] were entered as dependent variables. We modelled the Time x Condition interaction in this model, to assess involvement of brain areas in general emotion regulation, irrespective of valence. Next, we set up two LMEs including Condition (two levels: PCT, WLC) as between-subject fixed factor and Time (two levels: baseline, follow-up) as within-subject factor separately for the [Negative Down-Regulate > Negative Attend] contrast and for the [Positive Upregulation > Positive Attend] contrast.

In all models, subject was used to model a random intercept.

Statistical testing

FMRI effects were considered significant at $p<.05$, FWE corrected for multiple comparisons. We used 3dClustSim (36) to calculate the amount of voxels in a cluster needed to hold cluster-wise correction for multiple comparisons, with a group-level mask as input. As smoothness parameters, we calculated the average values across participants of their individual square-rooted residuals after first-level estimation. We used the cluster-extent threshold corresponding to the 2-sided NN1 clusters (i..e. voxels are clustered together if faces touch), at a voxel-wise p-threshold of 0.001 and a corrected alpha-level of 0.05. We repeated the 3dClustSim procedure restricted to a composite mask including our a priori regions of interest, based on the corresponding AAL (37) labels of the bilateral middle frontal gyrus, inferior frontal gyrus, medial frontal gyrus, cingulate gyrus, amygdala, and insula, using the WFU pick-atlas toolbox (38) implemented in SPM (see Supplement 9 for the composite mask). The resulting number of voxels (k) was then rounded-up and used as input to threshold the LME results (whole-brain: $k = 39$, effects occurring with the composite ROI: $k = 18$).

Supplement 9: Composite region of interest mask

The mask of the regions of interest (ROI) was created using the WFU Pickatlas (a toolbox implemented in SPM12) and based on the AAL atlas. It included labels corresponding to the bilateral middle frontal gyrus, frontal operculum, inferior frontal gyrus, superior medial gyrus, anterior cingulate gyrus, amygdala, superior parietal cortex and supplementary motor area.

AAL labels were:

- Frontal_Mid_L/R
- Frontal_Inf_Oper_L/R
- Frontal_Inf_Tri_L/R
- Frontal_Sup_Medial_L/R
- Cingulum_Ant_L/R
- Amygdala_L/R
- Parietal_Sup_L/R
- Supp_Motor_Area_L/R

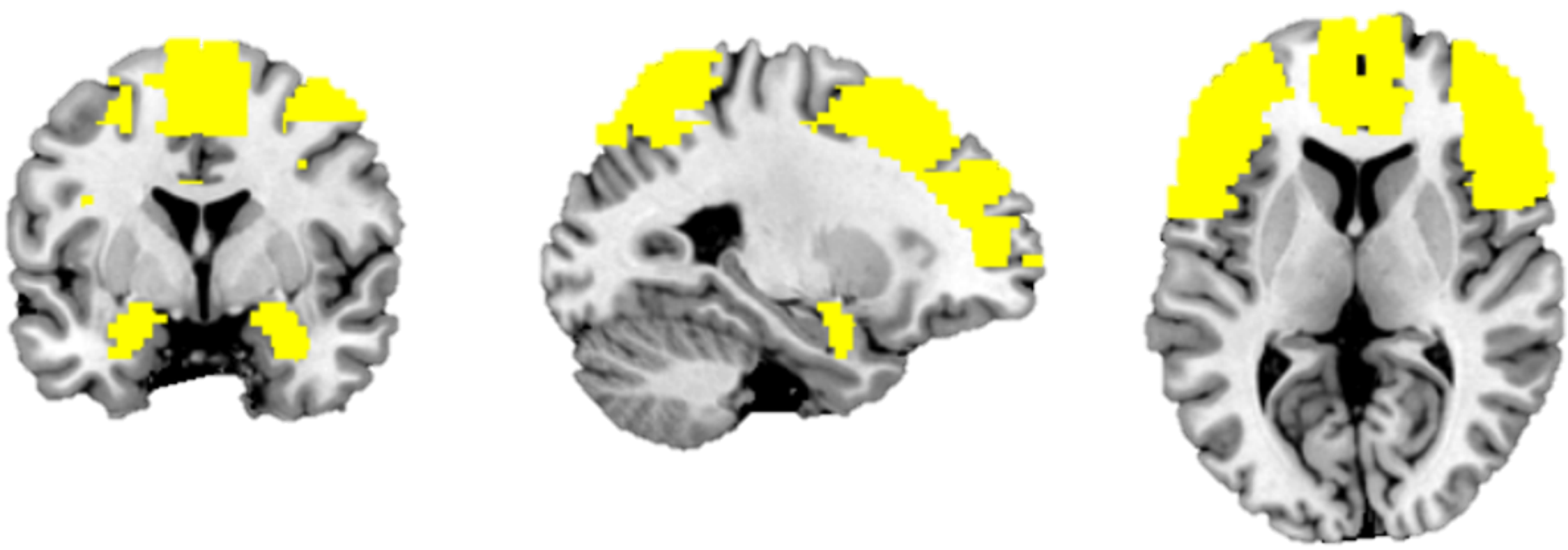

Figure S9.1. Composite mask

Supplement 10: Descriptives of outcome measures for the PCT and WLC groups

Supplemental Table 10.1 - Baseline and follow-up measurements: means, standard deviations (SD), minimum (min) and maximum (max) scores

	Descriptive Statistics			Baseline				Follow-up 1			
	Group	N	Missing	Mean	SD	Min	Max	Mean	SD	Min	Max
IDS30-SR - total score	Control	25	0	7.280	5.111	0.000	18.000	13.280	12.611	0.000	46.000
	Treatment	22	0	8.136	4.454	1.000	17.000	8.227	5.407	1.000	23.000
DAS - total score	Control	23	2	130.087	23.570	86.000	170.000	131.217	32.312	80.000	220.000
	Treatment	20	2	139.050	39.886	62.000	207.000	135.409	36.291	79.000	192.000
**Cognitive Reactivity**											
LEIDS-RR Total Score	Control	25	0	44.160	13.101	23.000	68.000	43.792	15.523	15.000	74.000
	Treatment	22	0	45.545	14.748	19.000	75.000	44.864	18.904	19.000	78.000
LEIDS-RR Hopelessness	Control	25	0	8.040	5.054	1.000	17.000	8.792	5.340	1.000	20.000
	Treatment	22	0	9.591	4.511	3.000	17.000	9.273	5.221	3.000	17.000
LEIDS-RR Acceptance	Control	25	0	2.200	2.723	0.000	9.000	1.750	1.894	0.000	6.000
	Treatment	22	0	1.318	1.615	0.000	6.000	1.227	2.069	0.000	8.000
LEIDS-RR Agression	Control	25	0	6.960	3.623	1.000	14.000	6.292	3.816	0.000	13.000
	Treatment	22	0	8.864	5.471	2.000	23.000	8.636	5.619	2.000	20.000
LEIDS-RR Control	Control	25	0	8.040	3.646	1.000	15.000	6.958	3.605	1.000	15.000
	Treatment	22	0	6.409	3.800	1.000	15.000	6.636	4.348	1.000	15.000
LEIDS-RR Rumination	Control	25	0	18.920	6.946	5.000	30.000	20.000	7.120	5.000	31.000
	Treatment	22	0	19.364	5.737	4.000	31.000	19.091	6.704	9.000	30.000
**Rumination**											
RSS - Total Score	Control	25	0	65.880	15.444	35.000	98.000	63.750	14.220	37.000	92.000
	Treatment	22	0	61.136	15.006	26.000	87.000	60.864	13.761	37.000	89.000
RSS - causal analysis	Control	25	0	17.800	3.488	10.000	24.000	17.292	3.141	11.000	21.000
	Treatment	22	0	16.864	4.313	7.000	23.000	16.636	3.360	10.000	23.000
RSS - understanding	Control	25	0	17.840	4.888	8.000	27.000	16.875	4.337	11.000	24.000
	Treatment	22	0	15.364	5.076	6.000	24.000	15.864	4.497	8.000	24.000
RSS - uncontrollability	Control	25	0	20.080	6.130	8.000	30.000	19.042	5.849	10.000	30.000
	Treatment	22	0	18.045	5.223	8.000	26.000	18.091	4.524	10.000	26.000
RPA - Total Score	Control	25	0	43.968	6.392	29.000	52.000	44.652	7.785	29.000	58.000
	Treatment	22	0	41.290	8.244	29.000	57.000	43.190	7.590	30.000	58.488
RPA - Self-focussed	Control	25	0	7.800	2.972	4.000	15.000	8.609	3.313	4.000	16.000
	Treatment	22	0	7.682	3.123	4.000	15.000	7.714	2.777	4.000	14.000
RPA - Dampening	Control	24	1	14.250	7.344	9.000	45.000	12.542	2.843	9.000	19.000
	Treatment	22	0	15.455	4.079	8.000	22.000	14.150	3.829	8.000	21.000
RPA - Emotion-focussed	Control	25	0	12.640	3.534	6.000	20.000	12.417	3.243	7.000	19.000
	Treatment	21	1	12.952	3.556	8.000	19.000	12.762	2.844	6.000	18.000
**Emotion regulation & Affect**											
ERQ - Cogn Reappraisal	Control	25	0	24.989	8.222	6.000	42.000	23.250	8.131	9.000	42.000
	Treatment	22	0	25.545	8.302	11.000	42.000	28.667	5.817	20.000	37.000
ERQ - Expr Suppression	Control	25	0	14.960	5.511	6.000	26.000	13.708	5.196	4.000	25.000
	Treatment	22	0	14.773	6.531	4.000	26.000	14.364	5.067	6.000	24.000
PANAS positive affect	Control	23	2	639.826	198.754	326.000	914.000	332.360	182.180	3.000	677.000
	Treatment	22	0	628.591	121.891	384.000	937.000	253.300	141.762	30.000	581.000
PANAS negative affect	Control	23	2	225.565	147.161	48.000	637.000	576.720	226.513	48.000	886.000
	Treatment	22	0	315.682	172.718	73.000	657.000	645.300	132.055	396.000	914.000
PANAS PA - variability	Control	23	2	46.217	31.392	0.000	93.000	53.440	27.644	0.000	100.000
	Treatment	22	0	64.091	18.728	18.000	100.000	59.400	20.798	24.000	100.000
PANAS PA - stability	Control	23	2	56.087	27.649	7.000	100.000	46.400	27.734	0.000	100.000
	Treatment	22	0	40.864	25.009	0.000	94.000	41.200	23.659	0.000	81.000
PANAS NA - variability	Control	23	2	60.565	27.655	0.000	94.000	62.160	22.291	0.000	100.000
	Treatment	22	0	71.591	13.308	46.000	100.000	64.000	19.059	36.000	100.000
PANAS NA - stability	Control	23	2	45.391	24.188	8.000	100.000	44.840	26.795	0.000	97.000
	Treatment	22	0	31.591	21.919	0.000	83.000	40.600	22.705	3.000	84.000
DOPS -	Control	25	0	72.069	12.092	51.190	94.190	64.975	16.128	28.000	86.860
consummatory/enjoy	Treatment	22	0	72.292	11.020	52.670	93.380	73.277	9.192	57.520	94.290
DOPS - motivation /	Control	25	0	65.031	14.076	40.000	91.360	62.129	17.506	17.430	92.930
anticipatory	Treatment	22	0	64.899	11.708	46.360	87.570	66.069	11.553	42.930	92.140
DOPS - general pleasure	Control	25	0	72.334	19.435	35.000	100.000	63.240	21.554	9.000	98.330
	Treatment	22	0	71.228	9.583	53.000	85.670	72.367	17.763	28.000	95.670
**Personality**											
BVAQ total score	Control	25	0	98.891	22.750	72.000	143.000	101.500	23.046	65.000	153.000
	Treatment	22	0	103.568	25.461	67.000	159.000	105.857	22.815	73.000	152.000
BVAQ Cognitive factor	Control	25	0	55.851	17.387	36.000	92.000	70.333	5.079	62.000	84.000
	Treatment	22	0	62.750	20.466	37.000	106.000	70.238	3.833	63.000	80.000
BVAQ Affective factor	Control	25	0	43.040	8.720	26.000	59.000	44.333	4.779	37.000	57.000
	Treatment	22	0	40.818	9.127	24.000	61.000	43.955	4.270	33.000	53.000
NEO-FFI Neuroticism	Control	25	0	34.580	6.872	21.000	45.000	35.633	8.396	17.000	54.000
	Treatment	22	0	37.764	4.904	29.000	47.000	38.136	5.874	28.000	50.000
NEO-FFI Extraversion	Control	25	0	37.774	7.014	27.000	49.000	37.750	6.661	25.000	51.000
	Treatment	22	0	37.188	7.863	16.000	50.000	37.136	8.043	19.000	50.000
NEO-FFI Conscientiousness	Control	25	0	39.374	6.781	25.000	50.000	38.875	8.629	26.000	58.000
	Treatment	22	0	42.864	6.875	27.000	55.000	42.000	6.640	33.000	53.000

Supplemental Table 10.2 - Baseline and follow-up measurements: means, standard deviations (SD), minimum (min) and maximum (max) scores

	Descriptive Statistics			Baseline				Follow-up 1			
	**Group**	**N**	**Missing**	**Mean**	**SD**	**Min**	**Max**	**Mean**	**SD**	**Min**	**Max**
**Emotional Reasoning Task**											
Negative - specific - no cue	Control	25	0	40.347	21.711	3.670	75.000	47.410	17.450	0.330	74.670
	Treatment	22	0	49.357	24.380	7.670	97.000	51.086	19.508	13.330	80.000
Negative - specific - cue	Control	25	0	44.900	21.762	9.000	84.000	41.282	21.975	2.400	76.670
	Treatment	22	0	52.695	23.886	8.800	91.830	47.444	21.353	14.000	80.000
Positive - specific - no cue	Control	25	0	56.073	16.688	7.000	80.830	57.810	13.927	26.830	92.330
	Treatment	22	0	58.724	16.004	28.170	92.500	61.330	16.167	23.250	84.500
Positive - specific - cue	Control	25	0	64.213	13.998	23.000	89.000	63.080	15.761	19.000	92.000
	Treatment	22	0	61.374	12.611	37.330	87.750	63.460	14.082	22.670	85.000
Negative - general - no cue	Control	25	0	23.354	18.735	0.000	65.000	24.646	17.595	0.330	59.670
	Treatment	22	0	29.592	21.262	0.330	73.830	32.698	23.687	0.330	68.670
Negative - general - cue	Control	25	0	25.427	15.949	0.000	47.170	27.097	19.422	0.000	64.670
	Treatment	22	0	33.809	20.820	0.000	81.330	35.023	22.929	2.170	63.330
Positive - general - no cue	Control	25	0	46.964	17.765	8.250	81.670	47.664	20.406	3.330	94.170
	Treatment	22	0	45.136	25.570	7.000	91.670	54.956	19.538	25.250	88.500
Positive - general - cue	Control	25	0	54.940	18.045	8.170	89.330	50.450	18.874	13.250	85.500
	Treatment	22	0	45.416	23.722	6.670	84.330	55.556	19.808	27.330	88.000
**ERT - affective and performance ratings**											
Neutral Attend - Affect	Control	24	1	56.750	7.607	46.670	82.330	56.476	7.534	45.670	73.670
	Treatment	21	1	57.223	9.056	46.000	83.670	57.818	9.354	49.670	88.000
Negative Attend - Affect	Control	24	1	45.653	11.408	22.670	83.330	45.396	13.047	25.000	72.670
	Treatment	21	1	42.715	14.946	18.670	74.670	50.666	15.257	19.000	87.000
Positive Attend - Affect	Control	24	1	63.902	8.738	50.000	87.000	63.905	6.722	50.000	76.000
	Treatment	21	1	64.793	10.384	50.000	87.000	63.633	11.380	50.000	91.330
Negative Regulate - Affect	Control	24	1	49.237	9.973	31.670	80.670	49.650	10.388	27.670	75.000
	Treatment	21	1	46.699	12.938	29.000	77.000	53.783	13.239	35.330	88.330
Positive Regulate - Affect	Control	24	1	65.596	11.326	50.000	95.000	61.714	7.748	50.000	75.000
	Treatment	21	1	67.190	9.832	51.330	87.330	65.216	11.514	50.000	91.000
Neutral Attend - Performance	Control	24	1	84.917	14.353	59.000	100.000	74.365	17.348	35.330	100.000
	Treatment	21	1	78.444	11.835	53.670	100.000	77.484	14.800	50.000	99.670
Negative Attend - Performance	Control	24	1	82.638	14.466	50.000	100.000	79.238	15.697	50.000	100.000
	Treatment	21	1	77.634	15.337	51.000	100.000	75.216	18.622	45.000	100.000
Positive Attend - Performance	Control	24	1	85.736	13.438	58.330	100.000	79.238	15.697	50.000	100.000
	Treatment	21	1	79.826	14.701	53.330	100.000	80.067	15.621	50.000	100.000
Negative Regulate - Performance	Control	24	1	75.958	15.170	42.000	94.000	62.429	16.759	39.330	89.330
	Treatment	21	1	68.778	14.844	41.330	100.000	74.316	15.510	50.000	100.000
Positive Regulate - Performance	Control	24	1	79.236	14.728	50.000	100.000	73.095	16.083	43.000	98.670
	Treatment	21	1	76.429	12.434	52.000	99.330	78.401	14.844	50.000	100.000
**ARDPEI**											
Engagement Bias - Negative - Short	Control	24	1	-79.140	164.911	-684.500	131.500	10.729	96.495	-161.500	209.500
	Treatment	20	2	3.045	133.041	-265.000	205.000	10.775	125.245	-174.500	304.500
Engagement Bias - Negative - Long	Control	24	1	18.720	286.762	-297.500	1.194.500	5.500	102.503	-192.500	212.000
	Treatment	20	2	41.750	99.249	-153.000	188.000	-19.700	128.630	-374.000	179.000
Engagement Bias - Positive - Short	Control	24	1	-113.360	156.192	-748.500	90.500	-53.708	75.660	-240.000	98.000
	Treatment	20	2	-80.568	90.524	-280.000	97.000	-43.750	102.959	-240.000	112.500
Engagement Bias - Positive - Long	Control	24	1	50.340	209.875	-246.500	765.500	50.396	141.595	-129.500	443.000
	Treatment	20	2	59.864	134.985	-132.500	437.500	48.950	123.816	-173.500	250.000
Disengagement Bias - Negative - Short	Control	24	1	22.100	106.491	-205.000	170.000	-60.167	110.037	-240.500	262.000
	Treatment	20	2	-21.295	107.355	-293.000	219.500	-48.100	140.841	-248.500	343.000
Disengagement Bias - Negative - Long	Control	24	1	14.440	185.225	-678.500	263.000	-40.688	177.076	-448.000	313.500
	Treatment	20	2	29.386	160.060	-189.000	359.000	42.450	103.466	-161.500	313.500
Disengagement Bias - Positive - Short	Control	24	1	73.000	161.506	-139.500	662.500	7.354	102.096	-163.000	286.000
	Treatment	20	2	56.023	160.808	-232.000	434.000	44.550	107.667	-81.500	303.500
Disengagement Bias - Positive - Long	Control	24	1	40.820	129.604	-192.500	262.500	1.604	102.572	-192.000	206.500
	Treatment	20	2	-2.250	177.227	-382.500	361.000	4.175	120.663	-208.500	272.000
**IAT**											
IAT score (D_600_ep_pt_weighted)	Control	24	1	0.571	0.521	-0.773	1.417	0.544	0.364	-0.488	1.195
	Treatment	22	0	0.513	0.371	-0.187	1.139	0.639	0.325	-0.041	1.084
IAT Explicit Negative Attributes	Control	25	0	31.725	19.415	0.000	81.800	35.352	15.487	6.600	63.800
	Treatment	22	0	32.855	15.216	4.000	55.800	26.589	14.960	3.800	52.200
IAT Explicit Positive Attributes	Control	25	0	65.133	17.701	21.400	94.600	64.088	17.471	21.600	88.600
	Treatment	22	0	61.009	16.331	33.800	91.000	68.895	15.929	35.200	96.600

Table S10.1 and 10.2. Outcome measures per condition per timepoint for all questionnaires & subscales (10.1) and tasks (10.2).

Supplement 11 Main task effects of the Emotion Regulation Task

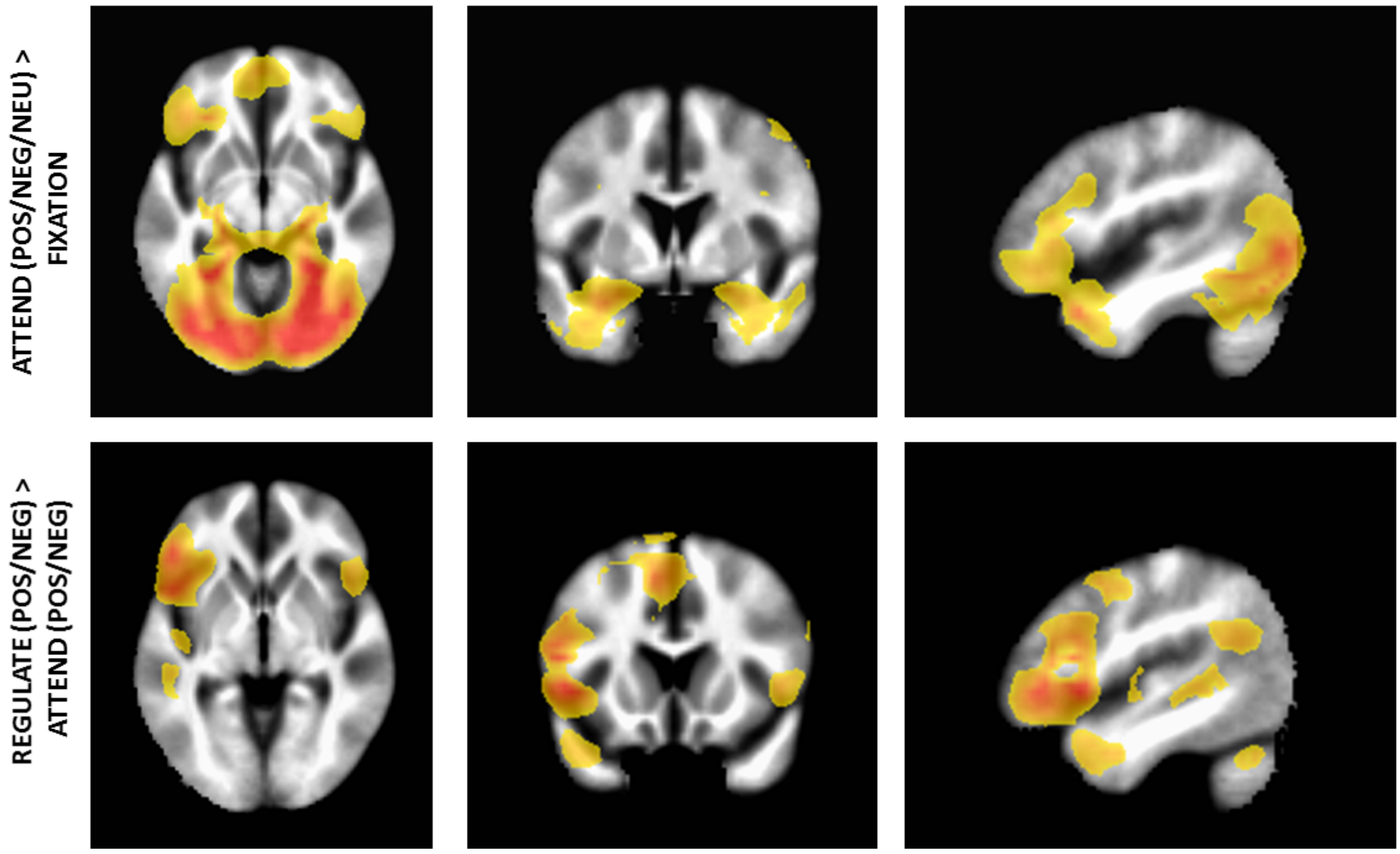

Figure S11.1. Main effects of task, for the contrasts 1) attend (positive, negative and neutral combined) versus fixation cross, and 2) regulate (positive and negative combined) versus attend (positive and negative combined) conditions.

Supplement references

1. van Kleef RS, Marsman J-BC, van Valen E, Bockting CLH, Aleman A, van Tol M-J (2022): Neural basis of positive and negative emotion regulation in remitted depression. *NeuroImage: Clinical* 34: 102988.

2. Kleef RSV, Bockting CLH, Valen EV, Aleman A, Marsman JBC, Tol MJV (2019): Neurocognitive working mechanisms of the prevention of relapse in remitted recurrent depression (NEWPRIDE): Protocol of a randomized controlled neuroimaging trial of preventive cognitive therapy. *BMC Psychiatry* 19: 409.

3. Bockting CLH (2009): *Preventieve Cognitive Training Bij Terugkerende Depressie*. Houten: Bohn Stafleu van Loghum. https://doi.org/10.1177/0146167204271418

4. Bockting C (2026): *Preventive Cognitive Therapy for Depression*. New York: The Guilford Press.

5. Luborsky L, Barber JP, Siqueland L, Johnson S, Najavits LM, Frank A, Daley D (1996): The Revised Helping Alliance Questionnaire (HAq-II) : Psychometric Properties. *J Psychother Pract Res* 5: 260–271.

6. Rush AJ, Carmody TJ, Ibrahim HM, Trivedi MH, Biggs MM, Shores-Wilson K, *et al.* (2006): Comparison of Self-Report and Clinician Ratings on Two Inventories of Depressive Symptomatology. *PS* 57: 829–837.

7. Rush AJ, Gullion CM, Basco MR, Jarrett RB, Trivedi MH (1996): The Inventory of Depressive Symptomatology (IDS): psychometric properties. *Psychol Med* 26: 477–486.

8. Van Roekel E, Vrijen C, Heininga VE, Masselink M, Bos EH, Oldehinkel AJ (2017): An Exploratory Randomized Controlled Trial of Personalized Lifestyle Advice and Tandem Skydives as a Means to Reduce Anhedonia. *Behavior Therapy* 48: 76–96.

9. Masselink M, van Roekel E, Heininga VE, Vrijen C, Oldehinkel A (2019): *Domains Of Pleasure Scale (DOPS): Assessing Pleasure across Domains*. Open Science Framework. https://doi.org/10.31219/osf.io/bu7z5

10. Crawford JR, Henry JD (2004): The positive and negative affect schedule (PANAS): construct validity, measurement properties and normative data in a large non-clinical sample. *The British journal of clinical psychology / the British Psychological Society* 43: 245–265.

11. Watson D, Clark LA, Tellegen A (1988): Development and validation of brief measures of positive and negative affect: the PANAS scales. *J Pers Soc Psychol* 54: 1063–1070.

12. First MB (Ed.) (2008): *Structured Clinical Interview for DSM-IV Axis I Disorders: SCID-I; Administration Booklet; [Compatible with Both DSM-IV and DSM-IV-TR]*, Clinician version, 9. [print.]. Arlington, Va: American Psychiatric Publ.

13. Lyketsos, C. G., Nestadt, G., Cwi, J., Heithoff, K. (n.d.): The Life Chart Interview: A standardized method to describe the course of psychopathology. *International Journal of Methods in Psychiatric Research* 4: 143–155.

14. Solis E, Antypa N, Conijn JM, Kelderman H, Van der Does W (2017): Psychometric properties of the Leiden Index of Depression Sensitivity (LEIDS). *Psychological Assessment* 29: 158–171.

15. Leuven Adaptation of the Rumination on Sadness Scale (L A RSS) (2002):

16. Raes F, Hermans D, Williams JMG, Bijttebier P, Eelen P (2008): A “triple W”-model of rumination on sadness: Why am I feeling sad, what’s the meaning of my sadness, and wish I could stop thinking about my sadness (but I can’t!). *Cognitive Therapy and Research* 32: 526–541.

17. Raes F, Daems K, Feldman GC, Johnson SL, Van Gucht D (2010): A Psychometric Evaluation of the Dutch Version of the Responses to Positive Affect Questionnaire. *PSYCHOL BELG* 49: 293.

18. Weissman A, Beck A (1978): Development and validation of the Dysfunctional Attitude Scale: a preliminary investigation. *Paper Presented at the Meeting of the Association for the Advancement of Behavior Therapy, Chicago, IL*. pp 1–33.

19. Arntz A, Rauner M, Hout M van den (1995): “If I feel anxious, there must be danger”: Ex-consqeuentia reasoning in inferring danger in anxiety disorders. *Behaviour Research and Therapy* 33: 917–925.

20. Van Kleef RS, Müller A, Van Velzen LS, Marie Bas-Hoogendam J, Van Der Wee NJA, Schmaal L, *et al.* (2023): Functional MRI correlates of emotion regulation in major depressive disorder related to depressive disease load measured over nine years. *NeuroImage: Clinical* 40: 103535.

21. Lang PJ, Bradley MM, Cuthbert BN, Greenwald M, Öhman A, Vaitl D, *et al.* (2008): International Affective Picture System (IAPS): Affective ratings of pictures and instruction manual International Affective Picture System (IAPS): Technical Manual and Affective Ratings. *International Affective Picture System (IAPS) International Affective Picture System (IAPS*.

22. Ioannidis CA, Siegling AB (2015): Criterion and incremental validity of the emotion regulation questionnaire. *Frontiers in Psychology* 6: 247.

23. Vorst HCM, Bermond B (2001): Validity and reliability of the Bermond–Vorst Alexithymia Questionnaire. *Personality and Individual Differences* 30: 413–434.

24. Grafton B, Southworth F, Watkins E, MacLeod C (2016): Stuck in a sad place: Biased attentional disengagement in rumination. *Emotion* 16: 63–72.

25. Glashouwer KA, Smulders FTY, de Jong PJ, Roefs A, Wiers RWHJ (2013): Measuring automatic associations: Validation of algorithms for the Implicit Association Test (IAT) in a laboratory setting. *Journal of Behavior Therapy and Experimental Psychiatry* 44: 105–113.

26. Mikels J a, Fredrickson BL, Larkin GR, Lindberg CM, Maglio SJ, Reuter-Lorenz P a (2005): Emotional category data on images from the International Affective Picture System. *Behavior research methods* 37: 626–630.

27. Glashouwer K a, Jong PJ de, Penninx BWJH (2012): Prognostic value of implicit and explicit self-associations for the course of depressive and anxiety disorders. *Behaviour Research and Therapy* 50: 479–486.

28. Elgersma HJ, Glashouwer KA, Bockting CLH, Penninx BWJH, de Jong PJ (2013): Hidden scars in depression? Implicit and explicit self-associations following recurrent depressive episodes. *Journal of Abnormal Psychology* 122: 951–960.

29. Tuijl LA van, Glashouwer KA, Elgersma HJ, Bockting CL, Penninx BW, Jong PJ de (2018): Depression recurrence after recovery_ Prognostic value of implicit and explicit self-depressed associations. *Behaviour Research and Therapy* 107: 76–82.

30. Berle D, Moulds ML (2013): Emotional Reasoning Processes and Dysphoric Mood: Cross-Sectional and Prospective Relationships. *PLoS ONE* 8. https://doi.org/10.1371/journal.pone.0067359

31. Berle D, Moulds ML (2013): An experimental investigation of emotional reasoning processes in depression. *British Journal of Clinical Psychology* 52: 316–329.

32. Verwoerd J, De Jong PJ, Wessel I, Van Hout WJPJ (2013): "If I feel disgusted, I must be getting ill": Emotional reasoning in the context of contamination fear. *Behaviour Research and Therapy* 51: 122–127.

33. Siegel JS, Power JD, Dubis JW, Vogel AC, Church JA, Schlaggar BL, Petersen SE (2014): Statistical improvements in functional magnetic resonance imaging analyses produced by censoring high-motion data points: Censoring High Motion Data in fMRI. *Hum Brain Mapp* 35: 1981–1996.

34. Cox RW (1996): AFNI: Software for Analysis and Visualization of Functional Magnetic Resonance Neuroimages. *Computers and Biomedical Research* 29: 162–173.

35. Chen G, Saad ZS, Britton JC, Pine DS, Cox RW (2013): Linear mixed-effects modeling approach to FMRI group analysis. *NeuroImage* 73: 176–190.

36. Cox RW, Chen G, Glen DR, Reynolds RC, Taylor PA (2017): FMRI clustering and false-positive rates. *Proceedings of the National Academy of Sciences of the United States of America* 114: E3370–E3371.

37. Tzourio-Mazoyer N, Landeau B, Papathanassiou D, Crivello F, Etard O, Delcroix N, *et al.* (2002): Automated Anatomical Labeling of Activations in SPM Using a Macroscopic Anatomical Parcellation of the MNI MRI Single-Subject Brain. *NeuroImage* 15: 273–289.

38. Maldjian JA, Laurienti PJ, Kraft RA, Burdette JH (2003): An automated method for neuroanatomic and cytoarchitectonic atlas-based interrogation of fMRI data sets. *NeuroImage* 19: 1233–1239.
